# Supplementary material for: Engineering a Seven Enzyme Biotransformation using Mathematical Modelling and Characterized Enzyme Parts
Source: ChemCatChem. 2019 Jul 4;11(15):3474–89. doi: 10.1002/cctc.201900646 (PMC6774274; doi:10.1002/cctc.201900646)
Supplement: Supplementary file 1 — Supplementary [file CCTC-11-3474-s001.pdf]

CHEMCATCHEM

Supporting Information

© Copyright Wiley-VCH Verlag GmbH & Co. KGaA, 69451 Weinheim, 2019

Engineering a Seven Enzyme Biotransformation using Mathematical Modelling and Characterized Enzyme Parts

William Finnigan, Rhys Cutlan, Radka Snajdrova, Joseph P. Adams, Jennifer A. Littlechild, and Nicholas J. Harmer*© 2019 The Authors. Published by Wiley-VCH Verlag GmbH & Co. KGaA. This is an open access article under the terms of the Creative Commons Attribution License, which permits use, distribution and reproduction in any medium, provided the original work is properly cited.

Supplementary Table and Figures

Contents

Supplementary Tables

Supplementary Table 1 – equilibrium constants for the reactions in the model 2

Supplementary Table 2 – Differential equations used in the modelling 4

Supplementary Figures

Supplementary Figures 1-8 – SDS-PAGE analysis of purification of proteins used in this study 5

Supplementary Figures 9-14 – activity of enzymes at different pH and temperature 9

Supplementary Figures 15-18 – understanding deviations from the model for esterase and CAR steps 13

Supplementary Figures 19-39 – Determination of catalytic parameters for enzymes 16

Supplementary Figure 40 – operational window for temperature and pH for the seven enzyme reaction 28

Supplementary Figure 41 – Sensitivity analysis of apADH and apADH-PTDH modelled reactions 29

Supplementary Figure 42 - Flow diagram for genetic algorithm 29

Supplementary Modelling: Alternative model incorporating magnesium

Supplementary Table 3 – Parameters in the alternative model 31

Supplementary Model Figures 43-46 - Comparisons of the model with an alternative model incorporating equilibriums for magnesium binding to ATP, ADP, PP_i and PolyP. 32

**Supplementary Table 1 – Equilibrium constants for the reactions in the model** Equilibrium constants were either taken from the literature, or calculated using thermodynamics. Equilibrium constants were used to justify reactions which were modelled as irreversible. Calculations for afEst2 and CAR are provided below.			
	**K**	**Modelled as Irreversible or Reversible**	**Ref**
**afEst2**	22	Rev	Calculated
**PTDH**	$1 * 10^{11}$	Irr	Woodyer 2003
**Ppiase**	$5.3 * 10^{3}$	Irr	Davies et al.1993
**CAR**	$7 * 10^{34}$	Irr	Calculated
**PAP**	NA	Rev	NA
**AK**	NA	Rev	NA
**apADH**	NA	Rev	NA

Calculation for afEst2

Methyl 4-methylbenzoate + water -> 4-methylbenzoate + methanol

ΔG_f for all of these (kJ mol^{-1}):
Water: -237 (Speight, 2017, Lange's Handbook of Chemistry, 17th ed., McGraw-Hill)
Methyl 4-methylbenzoate: -106 (www.chemeo.com)
4-methylbenzoate: -146 (www.chemeo.com)
Methanol: -179 (www.chemeo.com)

This gives ΔG_r^o = +18 kJ mol^{-1}.

To correct for the concentration of water, use the formula:

$$\Delta G_r \;=\; \Delta G_r^0 + R\,T\,ln\frac{c_{products}}{c_{reactants}}$$

Assuming a concentration of 2 mM (average) for other products and reactants here, and 56 M for water, this gives:

ΔG_r = 18 – 25.8 kJ mol^{-1} = -7.8 kJ mol^{-1}.

The equilibrium is given by:
$\Delta G = -R\,T \ln K$
So, $K = e^{-\Delta G / R\,T}$
K = 22
(note that when the concentrations in the above equation are set to the final concentrations observed in Figure 4, ΔG_r approximates to 0, as expected at equilibrium.)

Calculation for CAR
Benzoic acid + ATP + NADPH -> benzaldehyde + AMP + PP_i + $NADP^+$.

ΔG_f for all of these (kJ mol^{-1}):
Benzoic acid: -145.27
ATP: -2250.56
NADPH: 325.17
Benzaldehyde: 20.95
AMP: -514.41
PPi: -1936.41
NADP+: 260.5

(sources: benzoic acid and benzaldehyde – chemeo.com; others – ref [1])

The ΔG_r for the reaction is the sum of the ΔG_f for the products, minus the sum of the ΔG_f of the reactants.
This is:
20.95 -514.41 -1936.41+260.5 – (-145.27-2250.56+325.17) = -98.71 kJ/mol.

The reason why this is so favourable is that the acid supplies the oxygen. In a normal ATP hydrolysis, the hydroxide that has to be supplied has a ΔG_f of -159 kJ/mol, which makes the free energy gain much lower.

The equilibrium is given by:
$\Delta G = -R\ T \ln K$
So, $K = e^{-\Delta G / R\ T}$
Here, $K = e^{98.71 * 10^3 / 8.314 * 303}$
$K = 1.04 * 10^{17}$

Given this very large equilibrium constant, it seems sensible to treat this reaction as irreversible.

afEst2

$$\frac{dc_{Ester}}{dt} = -r1 + r2$$
$$\frac{dc_{Acid}}{dt} = r1 - r2$$

mpCAR

$$\frac{dc_{Acid}}{dt} = -r3$$
$$\frac{dc_{ATP}}{dt} = -r3$$
$$\frac{dc_{NADPH}}{dt} = -r3$$
$$\frac{dc_{Aldehyde}}{dt} = r3$$
$$\frac{dc_{Ppi}}{dt} = r3$$
$$\frac{dc_{AMP}}{dt} = r3$$
$$\frac{dc_{NADP+}}{dt} = r3$$

mpCAR-ttPPiase

$$\frac{dc_{Acid}}{dt} = -r3$$
$$\frac{dc_{ATP}}{dt} = -r3$$
$$\frac{dc_{NADPH}}{dt} = -r3$$
$$\frac{dc_{Aldehyde}}{dt} = r3 - r13$$
$$\frac{dc_{Ppi}}{dt} = r3 - r8$$
$$\frac{dc_{AMP}}{dt} = r3$$
$$\frac{dc_{NADP+}}{dt} = r3$$

mpCAR-ttPPiase-PTDH

$$\frac{dc_{Acid}}{dt} = -r3$$
$$\frac{dc_{ATP}}{dt} = -r3$$
$$\frac{dc_{NADPH}}{dt} = -r3 + r7$$
$$\frac{dc_{Aldehyde}}{dt} = r3 - r13$$
$$\frac{dc_{Ppi}}{dt} = r3 - r8$$
$$\frac{dc_{AMP}}{dt} = r3$$
$$\frac{dc_{NADP+}}{dt} = r3 - r7$$
$$\frac{dc_{PO3}}{dt} = -r7$$
$$\frac{dc_{PO4}}{dt} = r7$$

mpCAR-ttPPiase-PTDH-tnAK-tnPAP

$$\frac{dc_{Acid}}{dt} = -r3$$
$$\frac{dc_{ATP}}{dt} = -r3 + r11 - r12$$
$$\frac{dc_{ADP}}{dt} = r9 - r10 - r11 + r12$$
$$\frac{dc_{NADPH}}{dt} = -r3 + r7$$
$$\frac{dc_{Aldehyde}}{dt} = r7 - r13$$
$$\frac{dc_{Ppi}}{dt} = r3 - r8$$
$$\frac{dc_{AMP}}{dt} = r3 - r9 + r10 + r11 - r12$$
$$\frac{dc_{NADP+}}{dt} = r3 - r7$$
$$\frac{dc_{PO3}}{dt} = -r7$$
$$\frac{dc_{PO4}}{dt} = r7$$
$$\frac{dc_{PolyP}}{dt} = -r9 + r10$$

apADH

$$\frac{dc_{Aldehyde}}{dt} = -r4 + r5 - r13$$
$$\frac{dc_{Alcohol}}{dt} = r4 - r5$$
$$\frac{dc_{NADH}}{dt} = -r4 + r5$$
$$\frac{dc_{NAD+}}{dt} = r4 - r5$$

apADH-PTDH

$$\frac{dc_{Aldehyde}}{dt} = -r4 + r5 - r13$$
$$\frac{dc_{Alcohol}}{dt} = r4 - r5$$
$$\frac{dc_{NADH}}{dt} = -r4 + r5 + r6$$
$$\frac{dc_{NAD+}}{dt} = r4 - r5 - r6$$
$$\frac{dc_{PO3}}{dt} = -r6$$
$$\frac{dc_{PO4}}{dt} = +r6$$

afEst2-mpCAR-ttPPiase-PTDH-tnAK-tnPAP

$$\frac{dc_{Ester}}{dt} = -r1 + r2$$
$$\frac{dc_{Acid}}{dt} = r1 - r2 - r3$$
$$\frac{dc_{ATP}}{dt} = -r3 + r11 - r12$$
$$\frac{dc_{ADP}}{dt} = r9 - r10 - r11 + r12$$
$$\frac{dc_{NADPH}}{dt} = -r3 + r7$$
$$\frac{dc_{Aldehyde}}{dt} = r3 - r13$$
$$\frac{dc_{Ppi}}{dt} = r3 - r8$$
$$\frac{dc_{AMP}}{dt} = r3 - r9 + r10 + r11 - r12$$
$$\frac{dc_{NADP+}}{dt} = r3 - r7$$
$$\frac{dc_{PO3}}{dt} = -r7$$
$$\frac{dc_{PO4}}{dt} = r7$$
$$\frac{dc_{PolyP}}{dt} = -r9 + r10$$

mpCAR-ttPPiase-PTDH-tnAK-tnPAP-apADH

$$\frac{dc_{Acid}}{dt} = -r3$$
$$\frac{dc_{ATP}}{dt} = -r3 + r11 - r12$$
$$\frac{dc_{ADP}}{dt} = r9 - r10 - r11 + r12$$
$$\frac{dc_{NADPH}}{dt} = -r3 + r7$$
$$\frac{dc_{Aldehyde}}{dt} = r3 - r13 - r4 + r5$$
$$\frac{dc_{Alcohol}}{dt} = r4 - r5$$
$$\frac{dc_{Ppi}}{dt} = r3 - r8$$
$$\frac{dc_{AMP}}{dt} = r3 - r9 + r10 + r11 - r12$$
$$\frac{dc_{NADP+}}{dt} = r3 - r7$$
$$\frac{dc_{PO3}}{dt} = -r6 - r7$$
$$\frac{dc_{PO4}}{dt} = r6 + r7$$
$$\frac{dc_{PolyP}}{dt} = -r9 + r10$$
$$\frac{dc_{NADH}}{dt} = -r4 + r5 + r6$$
$$\frac{dc_{NAD+}}{dt} = r4 - r5 - r6$$

fEst2-mpCAR-ttPPiase-PTDH-tnAK-tnPAP-apADH

$$\frac{dc_{Ester}}{dt} = -r1 + r2$$
$$\frac{dc_{Acid}}{dt} = r1 - r2 - r3$$
$$\frac{dc_{ATP}}{dt} = -r3 + r11 - r12$$
$$\frac{dc_{ADP}}{dt} = r9 - r10 - r11 + r12$$
$$\frac{dc_{NADPH}}{dt} = -r3 + r7$$
$$\frac{dc_{Aldehyde}}{dt} = r3 - r13 - r4 + r5$$
$$\frac{dc_{Alcohol}}{dt} = r4 - r5$$
$$\frac{dc_{Ppi}}{dt} = r3 - r8$$
$$\frac{dc_{AMP}}{dt} = r3 - r9 + r10 + r11 - r12$$
$$\frac{dc_{NADP+}}{dt} = r3 - r7$$
$$\frac{dc_{PO3}}{dt} = -r6 - r7$$
$$\frac{dc_{PO4}}{dt} = r6 + r7$$
$$\frac{dc_{PolyP}}{dt} = -r9 + r10$$
$$\frac{dc_{NADH}}{dt} = -r4 + r5 + r6$$
$$\frac{dc_{NAD+}}{dt} = r4 - r5 - r6$$

Supplementary Table 2 – Differential equations used in the modelling. r1 to r12 refer to the rate equations in table 1 in the main text.

Supplementary Figures 1-8: SDS-PAGE analysis of purification of proteins used in this study

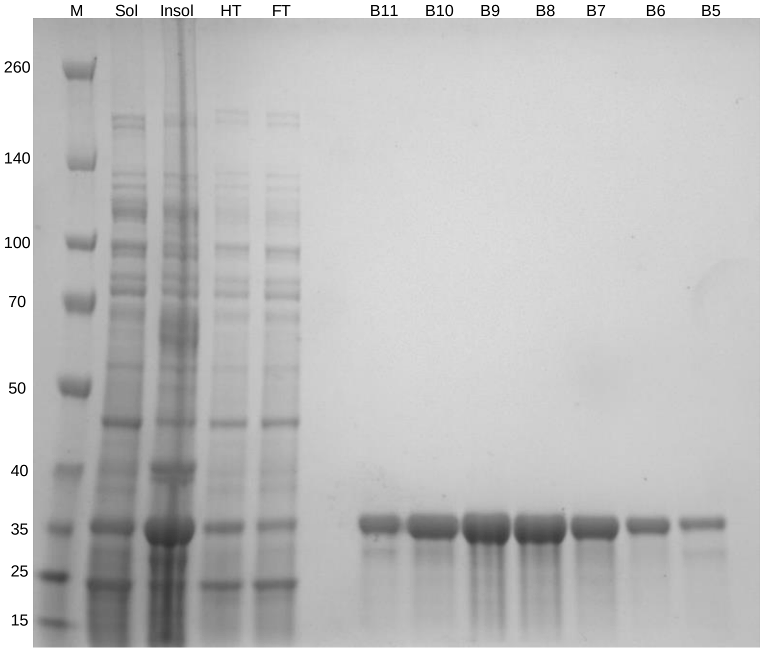

Supplementary Figure 1 - SDS-PAGE analysis of the AF-Est2 protein purification
Sol: Soluble fraction of cell lysate. Insol: Insoluble fraction of cell lysate. HT: Soluble lysate heat treated at 70 °C for 30 minutes before removal of precipitated proteins by centrifugation. FT: The flow through after loading the heat treated sample onto the nickel column. B11-B5: Fractions collected from the gel filtration column believed to be the purified AF-Est2 protein with an expected MW of 29 kDa.

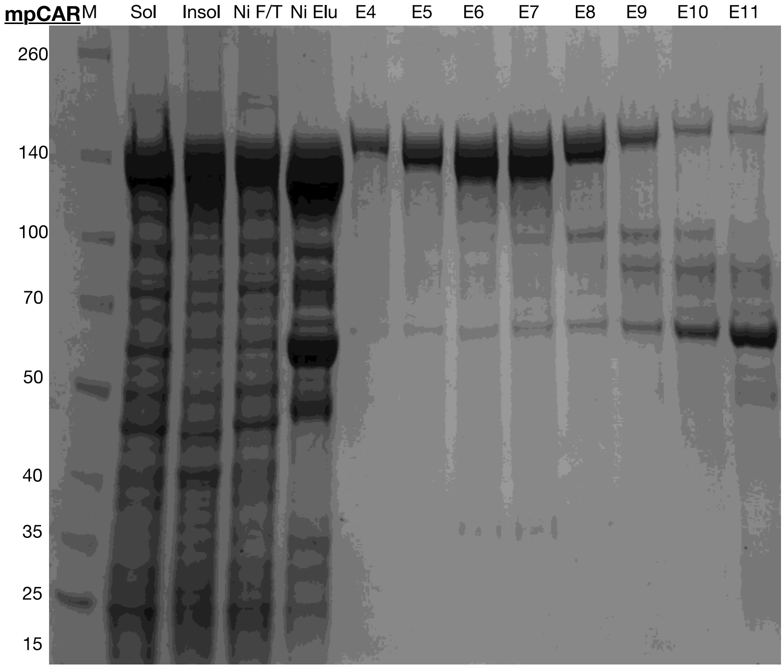

Supplementary Figure 2 - SDS-PAGE analysis of the mpCAR protein purification.
Sol: Soluble fraction of cell lysate. Insol: Insoluble fraction of cell lysate. Ni F/T: The flow through after loading the sample onto the nickel column. Ni Elu: Purified protein after the nickel purification step. E4-E11: Fractions collected from the gel filtration column believed to be the purified mpCAR protein with an expected MW of 128 kDa. Fractions E9 – E11 contained another unknown protein at approximately 60 kDa. These fractions were not pooled with the remaining fractions.

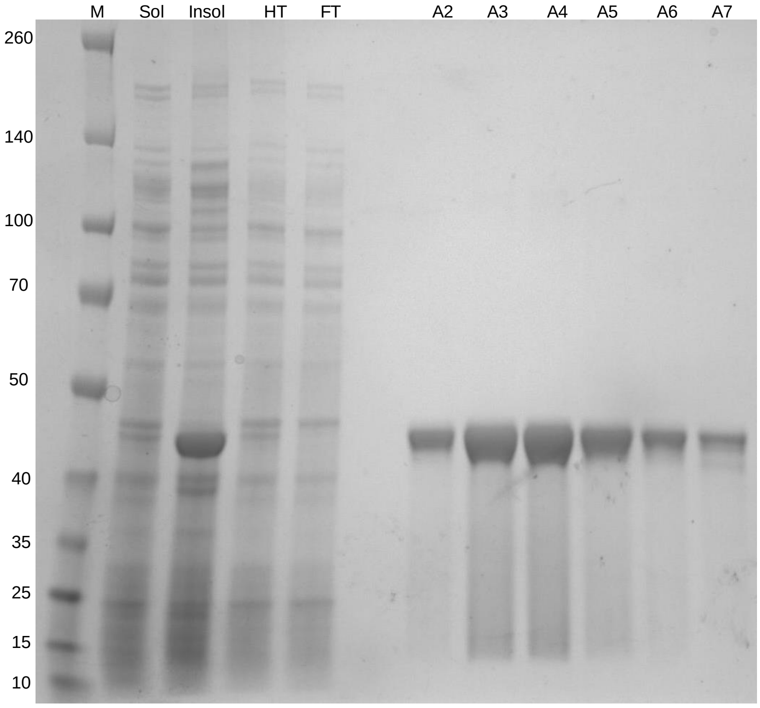

Supplementary Figure 3 - SDS-PAGE analysis of the ApADH protein purification.
Sol: Soluble fraction of cell lysate. Insol: Insoluble fraction of cell lysate. HT: Soluble lysate heat treated at 70 °C for 30 minutes before removal of precipitated proteins by centrifugation. FT: The flow through after loading the heat treated sample onto the nickel column. A2-A7: Fractions collected from the gel filtration column believed to be the purified ApADH protein with an expected MW of 41 kDa.

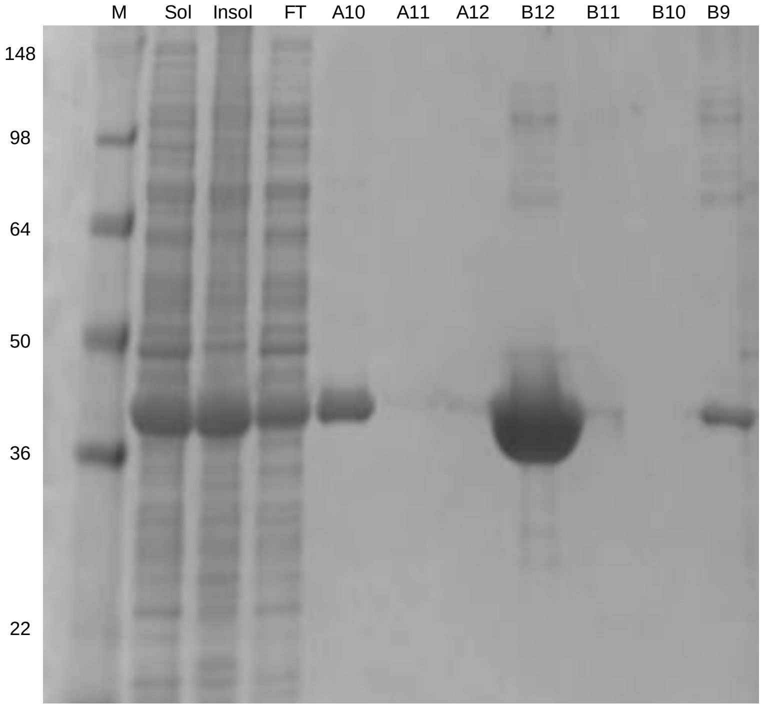

Supplementary Figure 4 - SDS-PAGE analysis of the PTDH protein purification.
Sol: Soluble fraction of cell lysate. Insol: Insoluble fraction of cell lysate. FT: The flow through after loading the sample onto the nickel column. A10-B9: Fractions collected from the gel filtration column believed to be the purified PTDH protein with an expected MW of 38.7 kDa. Very little protein was detected by SDS-PAGE in lanes A11, A112, B11 and B10, however there was likely a problem in running in the gel as there was an unavoidable delay between preparing the samples and running them. Samples at the extremes of the collected peak show pure protein so the entire peak was used.

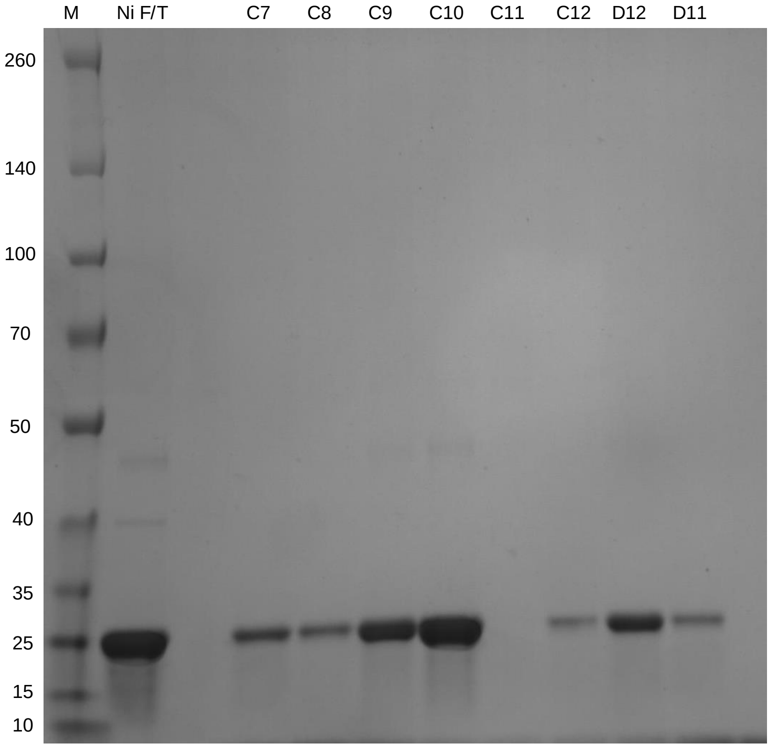

Supplementary Figure 5 - SDS-PAGE analysis of the ttPpiase protein purification.
Ni F/T: The flow through after loading the heat treated sample onto the nickel column. C7-D11: Fractions collected from the gel filtration column believed to be the purified ttPpiase protein with an expected MW of 21.8 kDa.

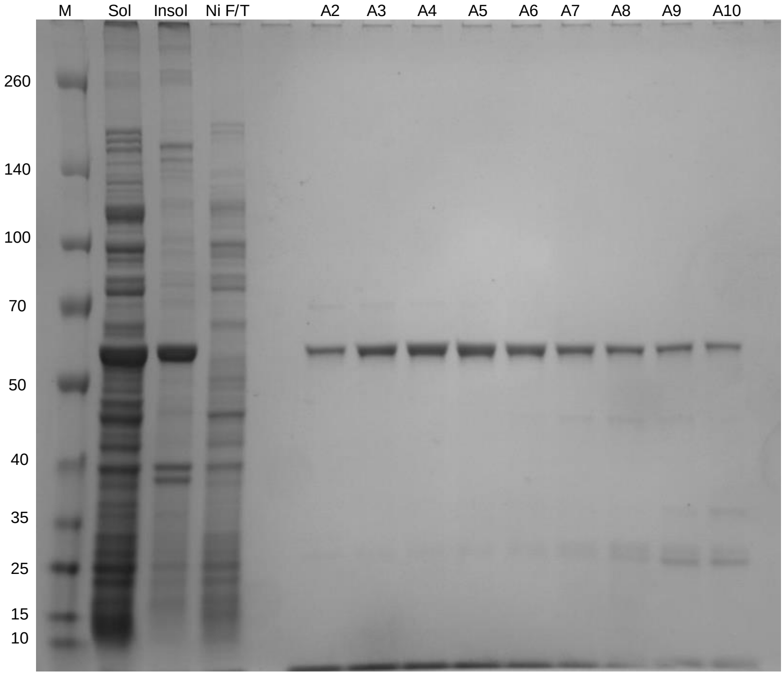

Supplementary Figure 6 - SDS-PAGE analysis of the tnPAP protein purification.
Sol: Soluble fraction of cell lysate. Insol: Insoluble fraction of cell lysate. Ni F/T: The flow through after loading the sample onto the nickel column. A2-A10: Fractions collected from the gel filtration column believed to be the purified tnPAP protein with an expected MW of 61.1 kDa.

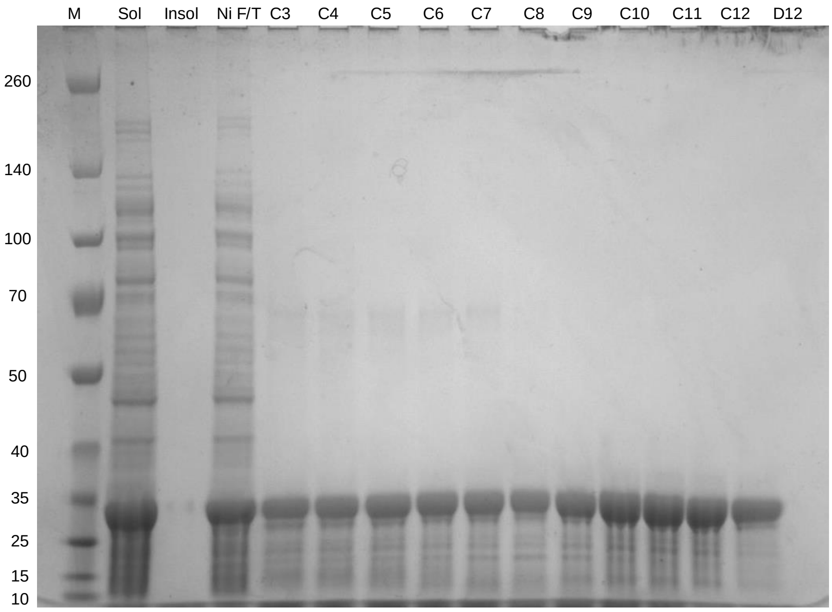

Supplementary Figure 7 - SDS-PAGE analysis of the tnAK protein purification.
Sol: Soluble fraction of cell lysate. Insol: Insoluble fraction of cell lysate. Ni F/T: The flow through after loading the sample onto the nickel column. C3-D12: Fractions collected from the gel filtration column believed to be the purified tnPPT protein with an expected MW of 27.6 kDa.

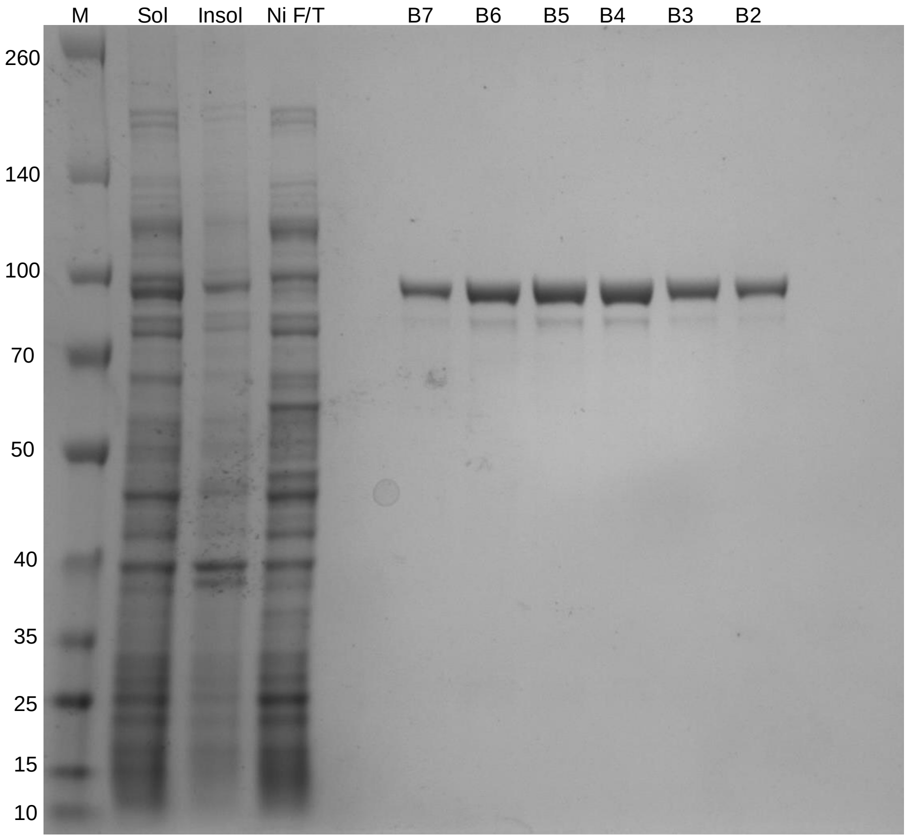

Supplementary Figure 8 - SDS-PAGE analysis of the tePPK protein purification.
Sol: Soluble fraction of cell lysate. Insol: Insoluble fraction of cell lysate. Ni F/T: The flow through after loading the sample onto the nickel column. B7-B2: Fractions collected from the gel filtration column believed to be the purified tePPK protein with an expected MW of 87.5 kDa.

Supplementary Figures 9-14: Activity of enzymes at different pH and temperature

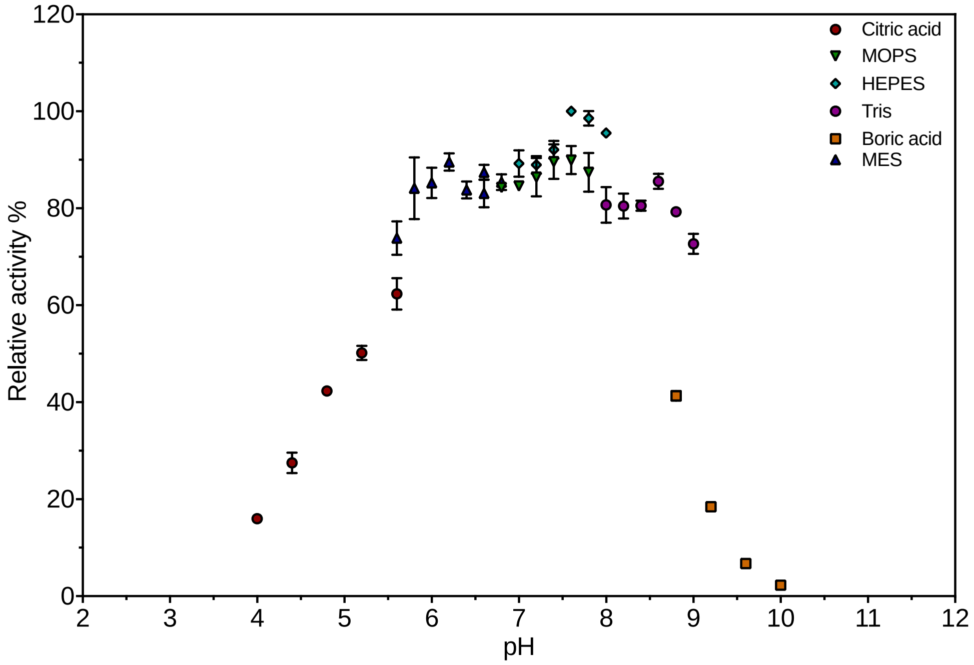

Supplementary Figure 9 – PTDH activity at different pH values

Activity of PTDH at various pH values, relative to the maximum activity detected. Various buffers were used to cover the range of pH values as indicated.

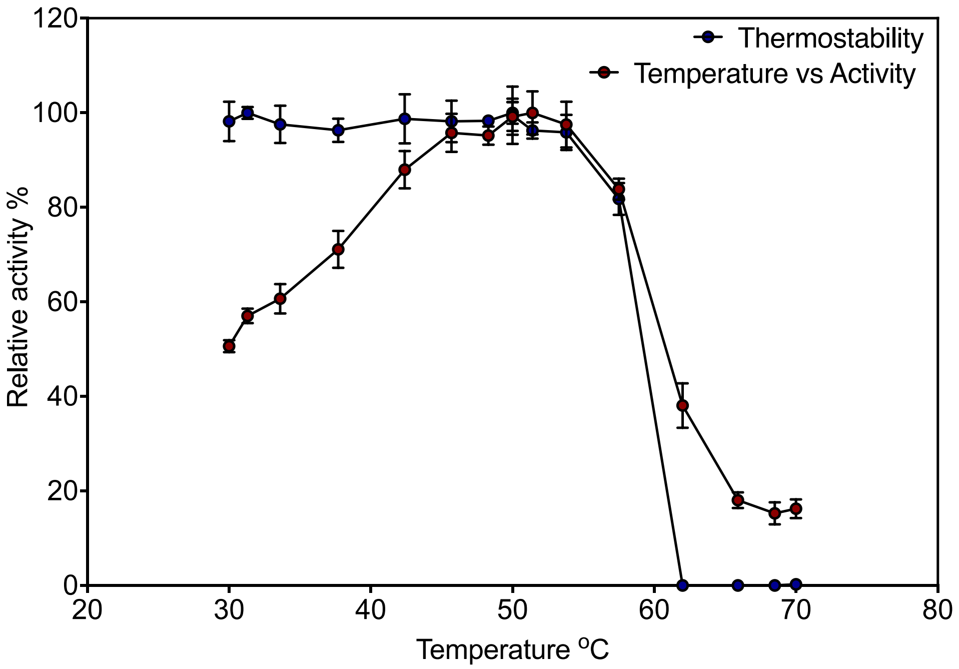

Supplementary Figure 10 – PTDH thermostability and temperature vs activity
Red circles show PTDH activity at temperature, relative to the maximum activity at 51.4 °C. Blue circles show residual activity after incubating PTDH at various temperatures for 30 minutes, relative to a control kept on ice.

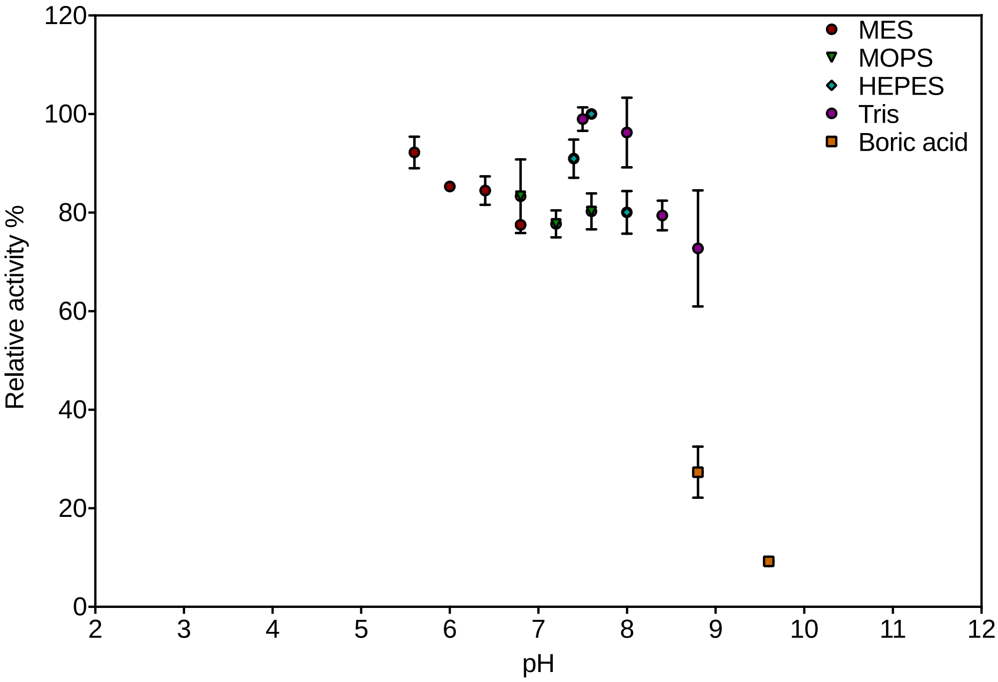

Supplementary Figure 11 – tnPAP activity at different pH values
Activity of tnPAP at various pH values, relative to the maximum activity detected. Various buffers were used to cover the range of pH values as indicated.

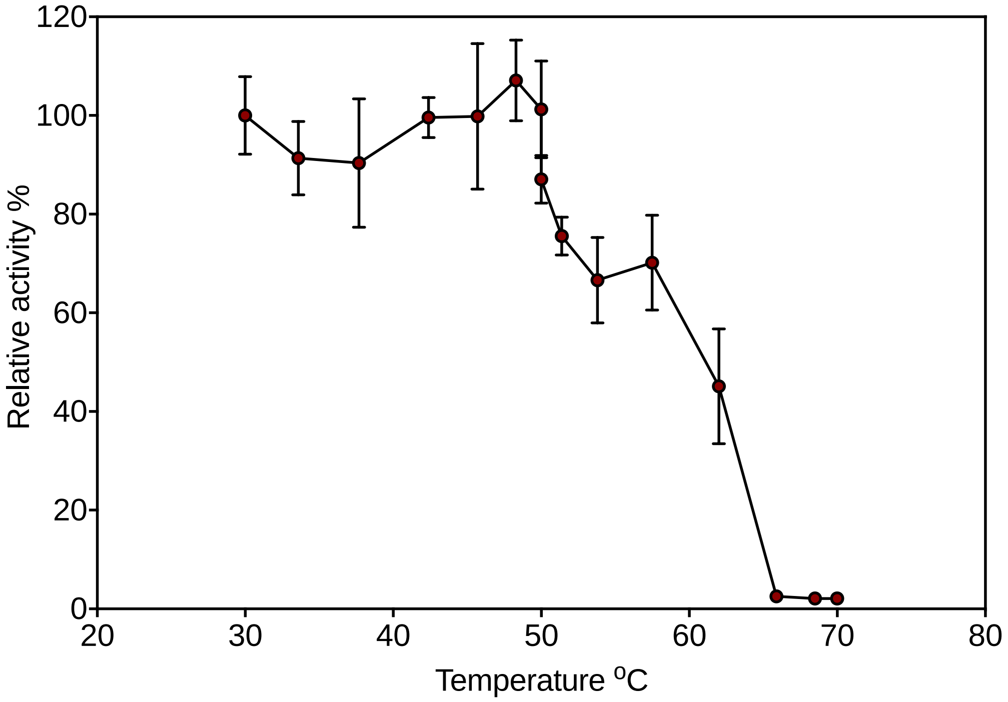

Supplementary Figure 12 – tnPAP thermostability

Residual activity after incubating tnPAP at various temperatures for 30 minutes, relative to a control kept on ice.

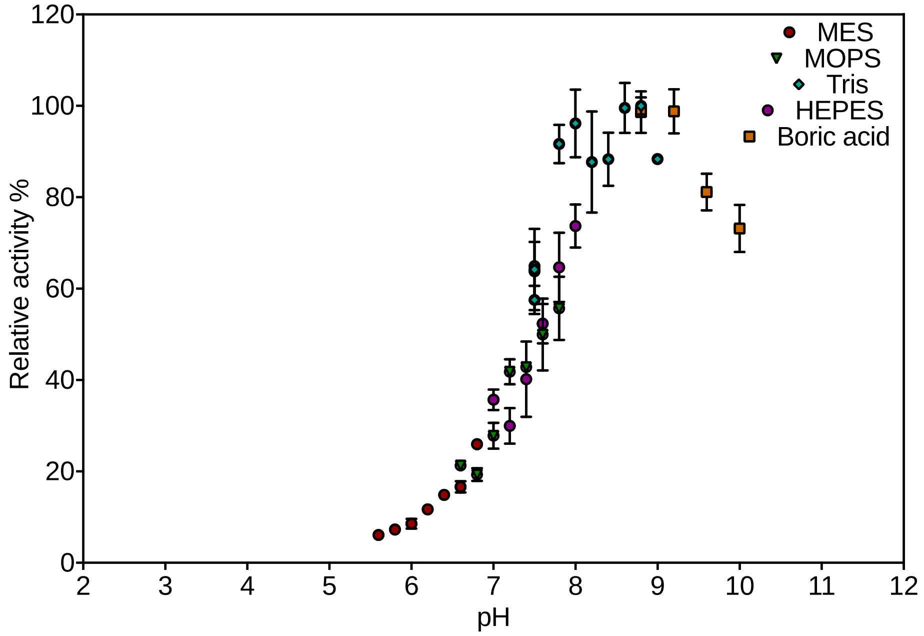

Supplementary Figure 13 – ttPPiase activity at pH

Activity of ttPPiase at various pH values, relative to the maximum activity detected. Various buffers were used to cover the range of pH values as indicated.

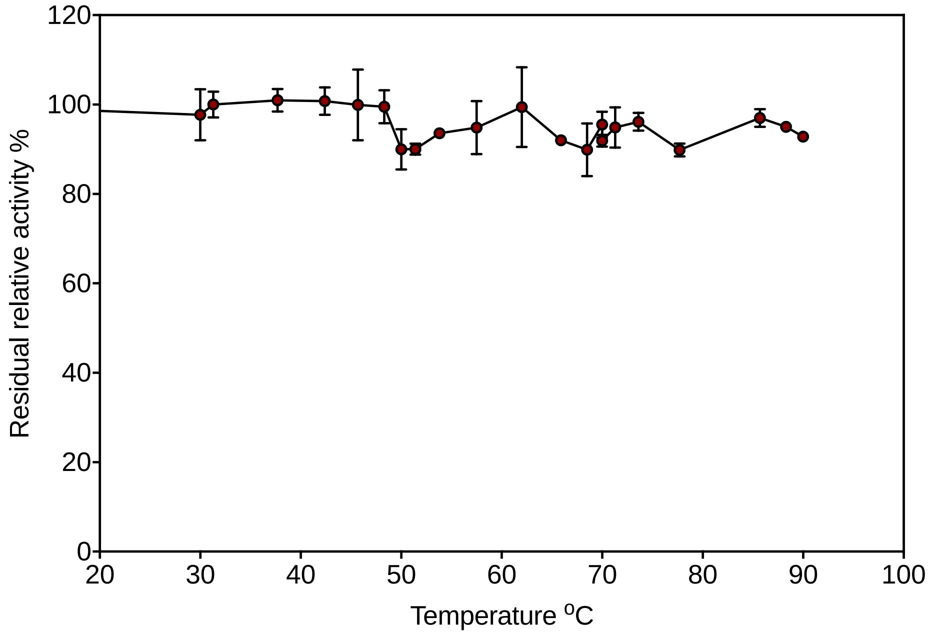

Supplementary Figure 14 - ttPPiase thermostability
Residual activity after incubating ttPPiase at various temperatures for 30 minutes, relative to a control kept on ice.

Supplementary Figures 15-18: Understanding deviations from the model for esterase and CAR steps

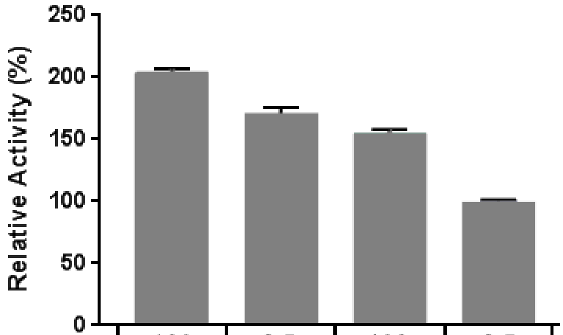

Tris concentration (mM)	100	2.5	100	2.5
Auxiliary reagents	+	+	-	-
Mean (%)	203.9	170.7	155.1	100

	F Value.	P Value.
Tris - HCl.	166.17	$3.8*10^{-11}$
Auxiliary reagents.	319.01	$9.3*10^{-14}$
Tris - HCl:Addtives.	23.62	$9.5*10^{-05}$

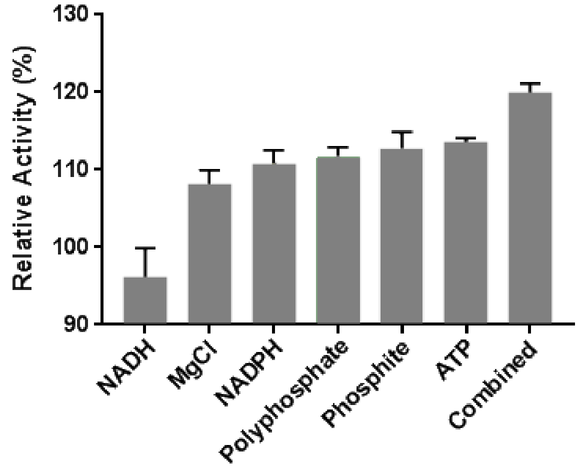

Supplementary Figure 15 – The rate of afEst2 increases upon the addition of final reaction components. The afEST2 enzyme assay (Sayer et al., 2017) was performed in conditions replicating the differences between initial assay and final assay conditions. The reaction was initiated by the addition of 500 µM of substrate (4-nitrophenyl-butyrate). The production of 4-nitrophenol was observed at 405 nm. A. Effect of auxiliary reactants and buffer. The reaction was performed in the presence of 2.5 or 100 mM Tris-HCl pH 7.5, and with or without the auxiliary reactants from the final assay (NADH (500 µM), MgCl2 (20,000 µM), NADPH (500 µM), polyphosphate (6,000 µM), phosphite (20,000 µM) or ATP (1,250 µM)). An increase in buffer concentration causes a 55% increase in rate (p < 10-10), whilst the addition of auxiliary reagents causes a 70% increase in rate (p < 10-13). The addition of the auxiliary reagents to the high buffer condition causes a further increase of ~30%, implying that there is a less than additive effect of the two changes (p < 10-4; all statistics calculated using a two-way ANOVA with Tukey's post-hoc determination). Experiments were performed in sextuplet. B. Effects of individual auxiliary reactants. The reaction was performed in the presence of each auxiliary reactant alone and compared to control, in 100 mM Tris-HCl pH 7.5. The afEST2 rate increased compared to the control for all of the auxiliary reactants tested, with the exception of NADH. The sum of these increases was considerably more than the increase seen for the combination of all reagents, implying that the effects of these are not necessarily specific. This further implies that most likely the effect of these treatments is ionic stabilization of the protein rather than a specific interaction. Experiments were performed in triplicate. Error bars show SEM.

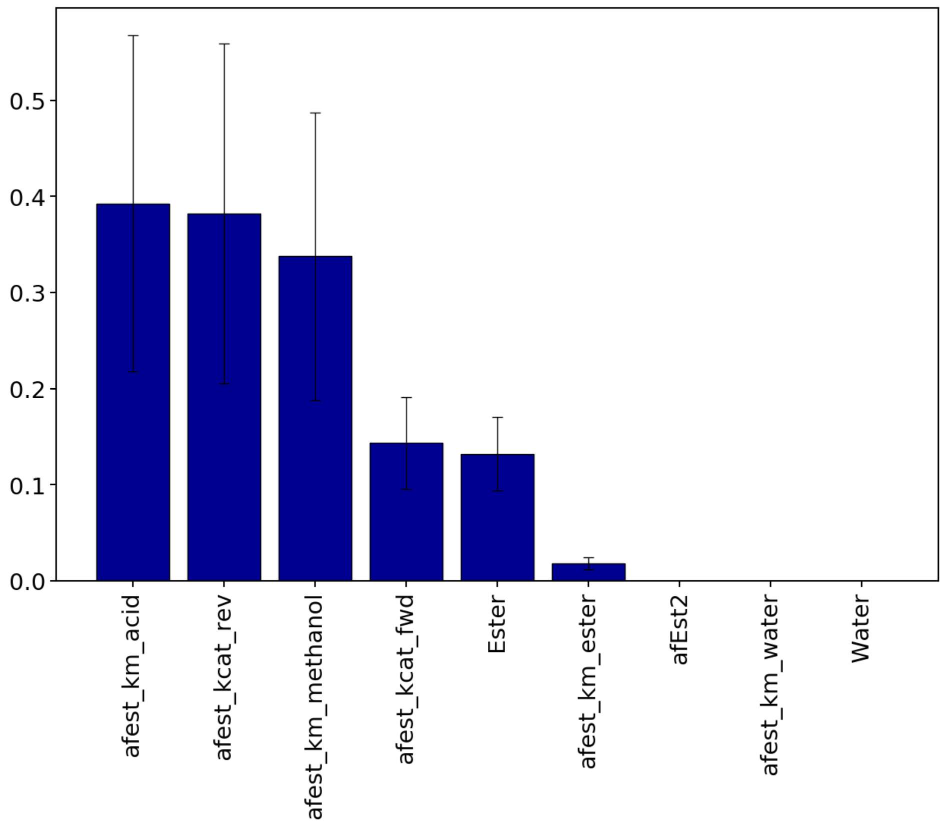

Supplementary Figure 16 - Sensitivity analysis of the reversible afEst2 reaction.
The total sensitivity indices (ST) are shown which take into account 1st order and all other interactions. Sensitivity is in reference to the uncertainty in the final methyl-*p*-toluate concentration. Error bars show the 95 % confidence intervals. The sum of all sensitivity indices' should equal 1.

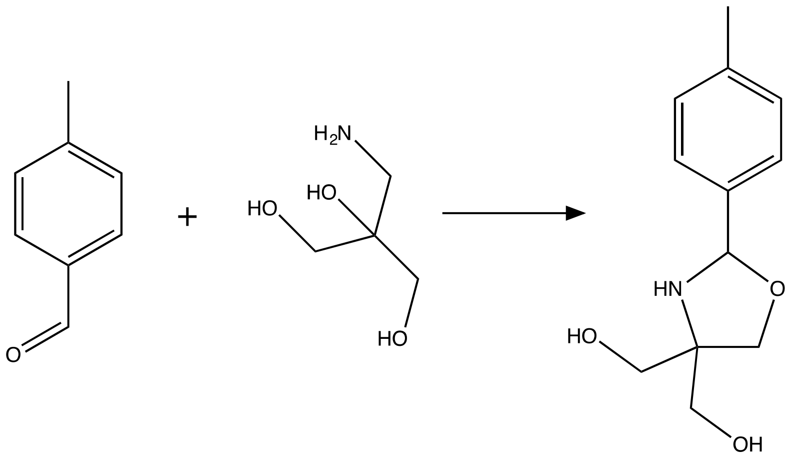

Supplementary Figure 17 – Proposed side reaction between 4-methylbenzalde and Tris
The proposed side reaction yields a product with an exact mass of 223.1.

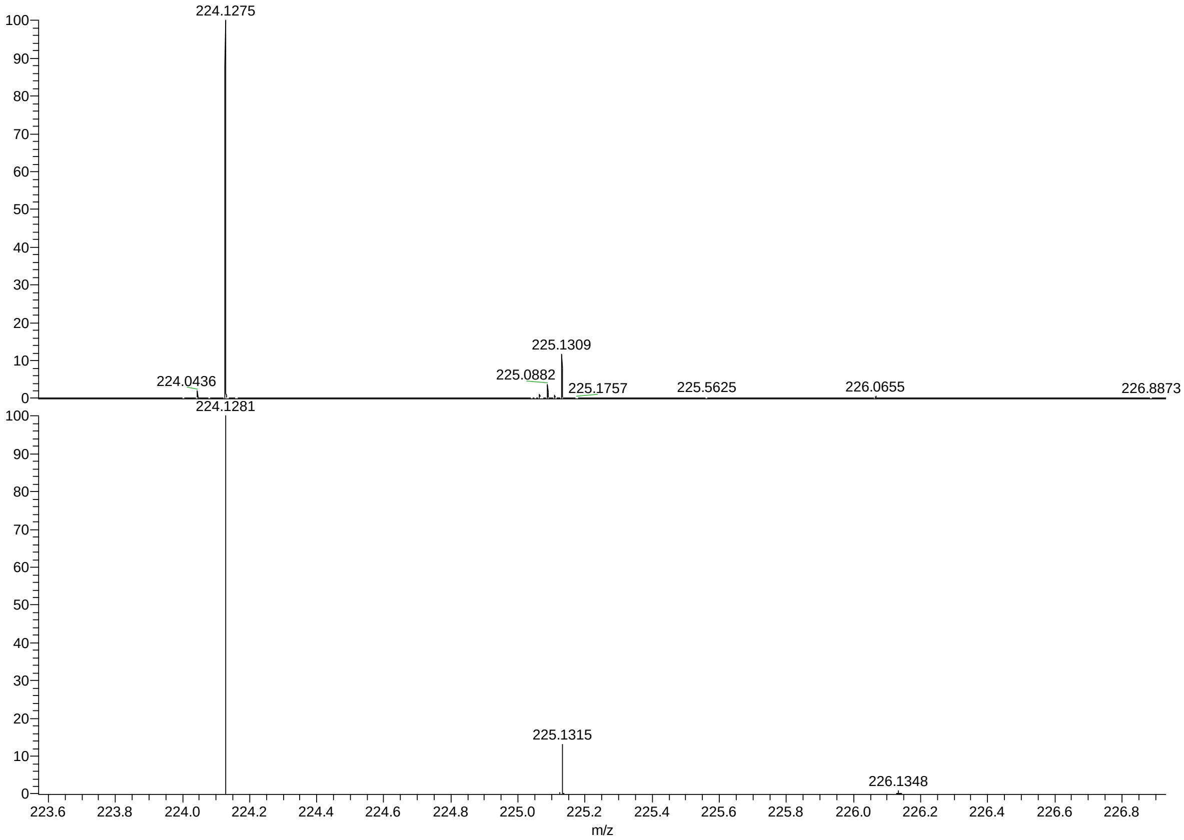

Supplementary Figure 18 – Mass spec analysis of a reaction between 4 mM 4-methylbenzaldehyde and 100 mM Tris.

The recorded HRMS and the theoretical isotopic pattern for the product proposed in Figure 5 – Supplementary Figure 1. 4 mM 4-tolualdehyde was incubated in 100 mM Tris-HCl pH 7.5 overnight at room temperature, and a sample taken. The recorded data and the theoretical isotopic pattern for the proposed and the produced products are in accordance with each other.

Supplementary Figures 19-39: Determination of catalytic parameters for enzymes

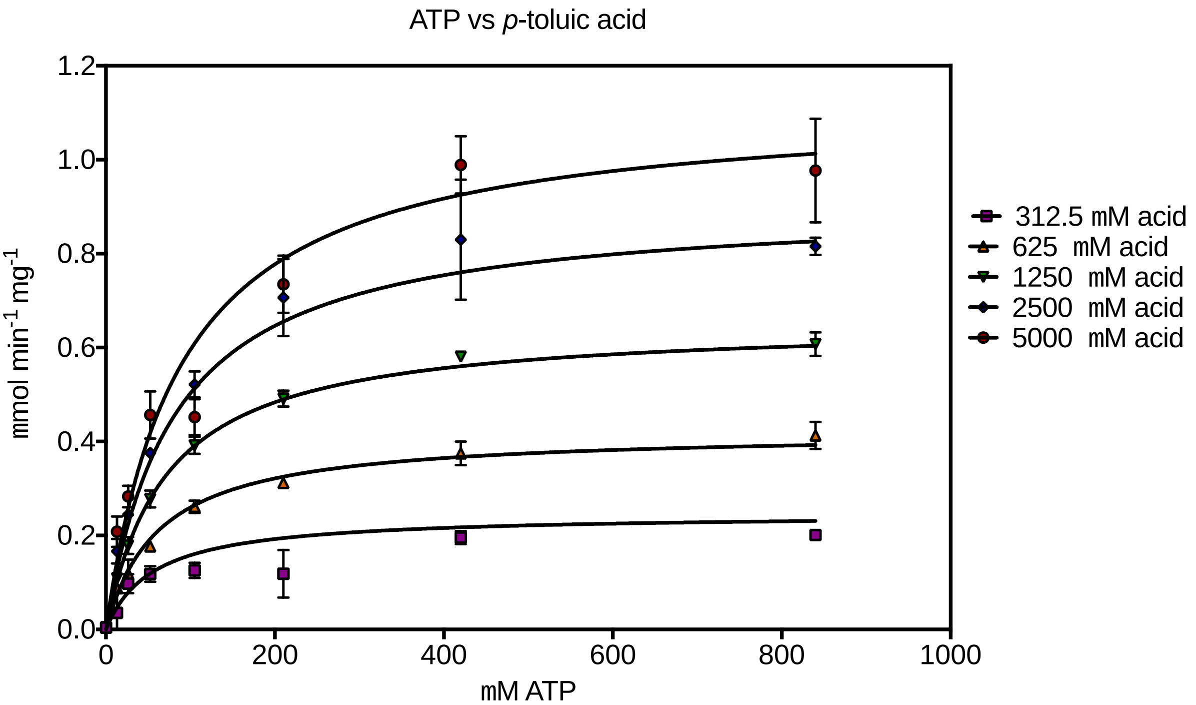

Supplementary Figure 19 - The kinetics of mpCAR with varying concentrations of ATP and 4-toluic acid. Data were fitted best to the equation for a steady-state sequential reaction.

**Supplementary Figure 20 – Parameters calculated fitting kinetics of mpCAR with varying concentrations of ATP and 4-toluic acid to a sequential steady state equation.** $v = V_{max} \cdot \frac{[A]\cdot[B]}{(K_I^A \cdot K_M^B)+(K_M^A \cdot [B])+(K_M^B \cdot [A])+([A]\cdot[B])}$	
**Best-fit values**	
V_{MAX} (µmol / min / mg)	1.5
K_I ATP (µM)	50
K_M Acid (µM)	1,500
K_M ATP (µM)	100
**Std. Error**	
V_{MAX} (µmol / min / mg)	0.07
K_I ATP (µM)	20
K_M Acid (µM)	200
K_M ATP (µM)	10

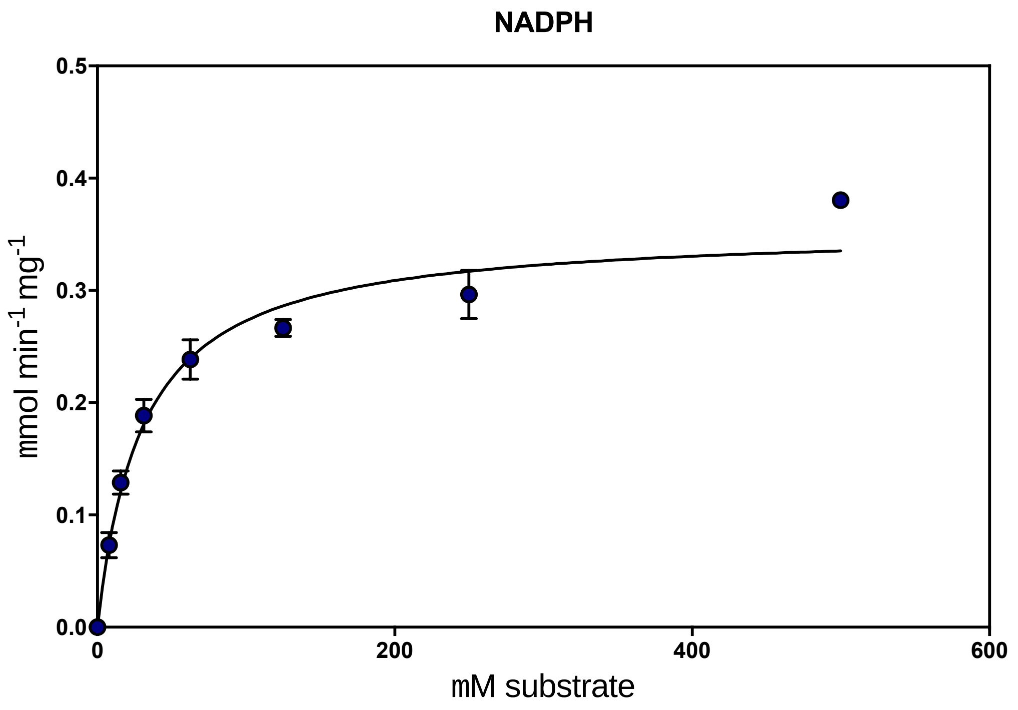

Supplementary Figure 21 – The kinetics of mpCAR with varying concentrations NADPH. Data have been fitted to the Michaelis-Menten equation.

**Supplementary Figure 22 -Parameters calculated fitting kinetics of mpCAR with varying concentrations of NADPH to the Michaelis-Menten equation.**	
**Best-fit values**	
V_{MAX} (µmol / min / mg)	0.35
K_M NADPH (µM)	30
**Std. Error**	
V_{MAX} (µmol / min / mg)	0.01
K_M NADPH (µM)	4

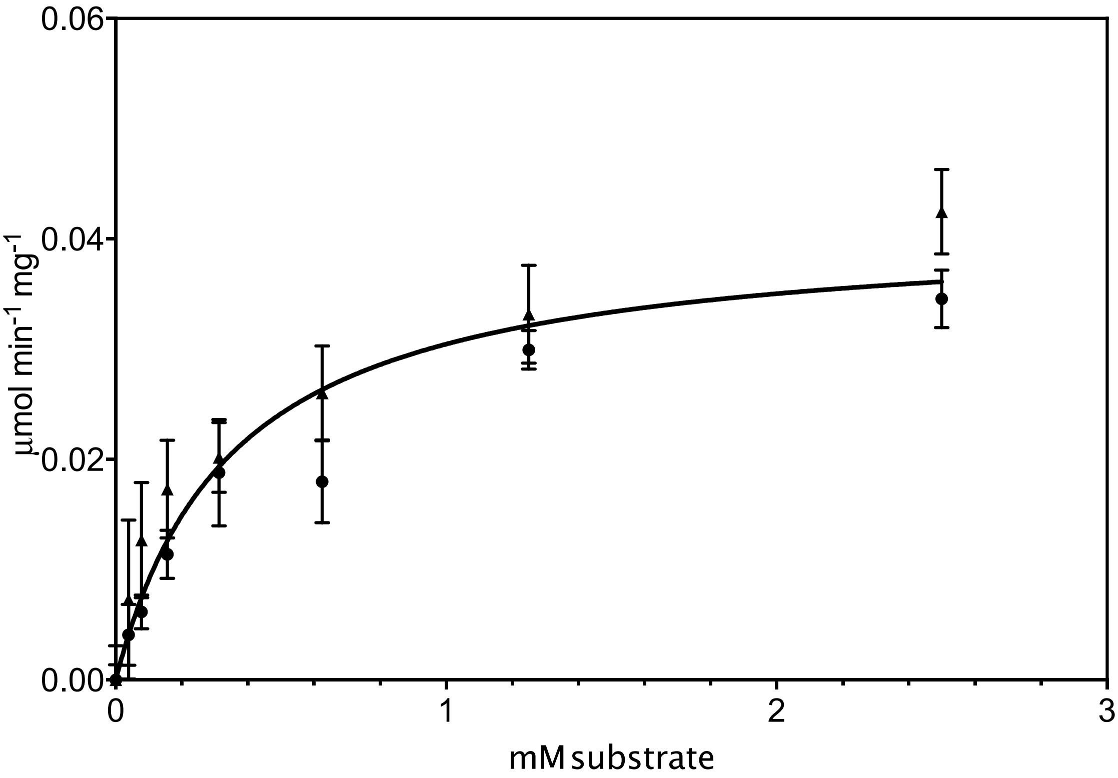

Supplementary Figure 23 - The kinetics of apADH in the reductive direction at 30 °C, pH 7.5 with varying concentrations of 4-tolualdehyde.
Two experiments were carried out shown as triangles and circles, with the data combined for fitting to the Michaelis-Menten equation.

**Supplementary Figure 24 - Parameters calculated fitting kinetics of apADH in the reductive direction at 30 °C, pH 7.5, with varying concentrations of 4-tolualdehyde to the Michaelis-Menten equation.**	
**Best-fit values**	
V_{MAX} (µmol / min / mg)	0.041
K_M *p*-tolualdehyde (µM)	350
**Std. Error**	
V_{MAX} (µmol / min / mg)	0.002
K_M *p*-tolualdehyde (µM)	60

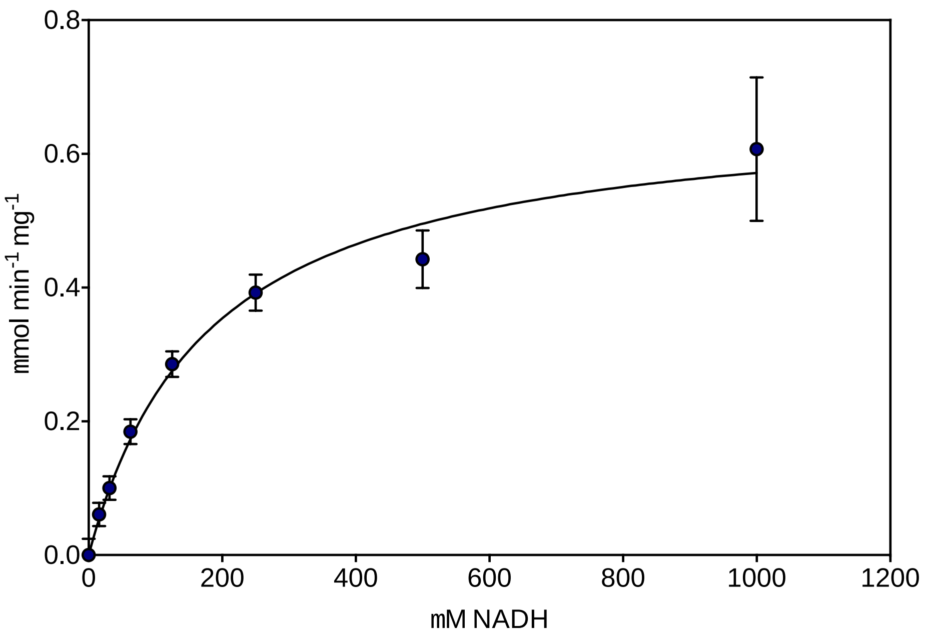

Supplementary Figure 25 - The kinetics of apADH in the reductive direction at 70 °C, pH 7.5 with varying concentrations of NADH.
Data have been fitted to the Michaelis-Menten equation.

Supplementary Figure 26 - Parameters calculated fitting kinetics of apADH in the reductive direction at 70 °C, pH 7.5, with varying concentrations of NADH, to the Michaelis-Menten equation.
Only K_M used in this work.

**Best-fit values**	
V_{MAX} (µmol / min / mg)	0.68
K_M NADH (µM)	180
**Std. Error**	
V_{MAX} (µmol / min / mg)	0.04
K_M NADH (µM)	30

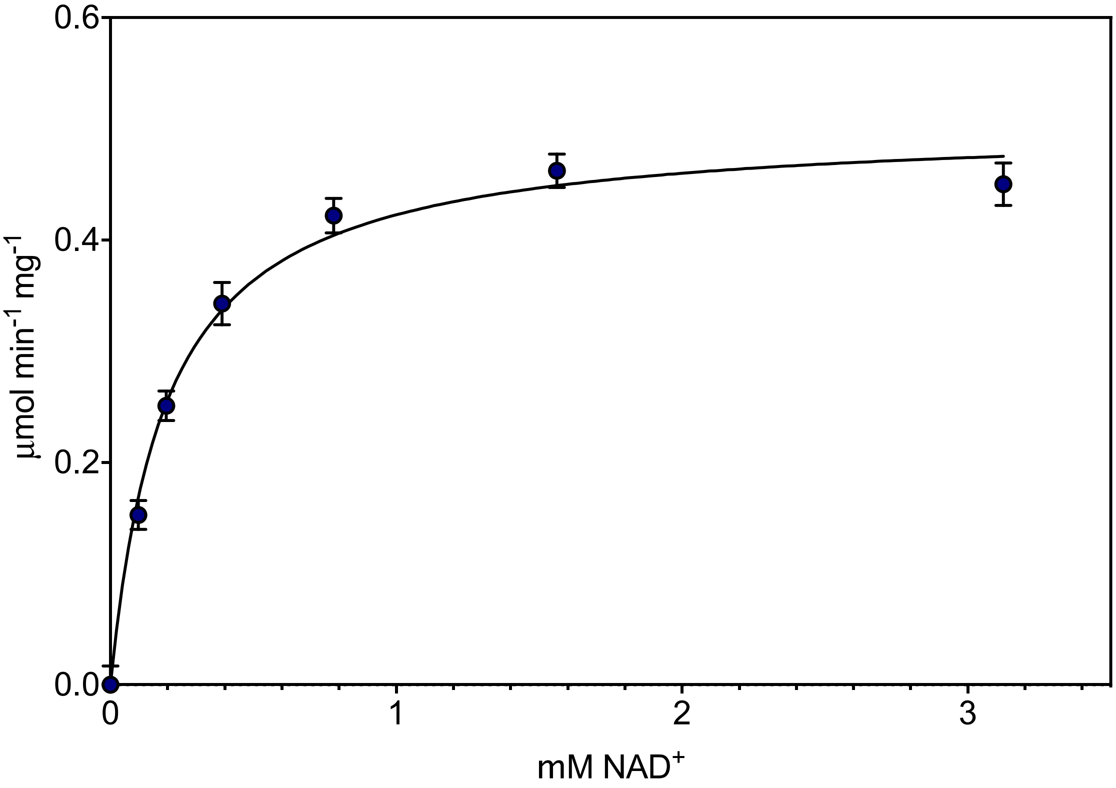

Supplementary Figure 27 - The kinetics of apADH in the oxidative direction at 70 °C, pH 7.5 with varying concentrations of NAD$^+$.
Data have been fitted to the Michaelis-Menten equation.

**Supplementary Figure 28 - Parameters calculated fitting kinetics of apADH in the oxidative direction at 70 °C, pH 7.5, with varying concentrations of NAD$^+$, to the Michaelis-Menten equation.** Only K_M used in this work.	
**Best-fit values**	
V_{MAX} (µmol / min / mg)	0.50
K_M NAD$^+$ (µM)	195
**Std. Error**	
V_{MAX} (µmol / min / mg)	0.01
K_M NAD$^+$ (µM)	16

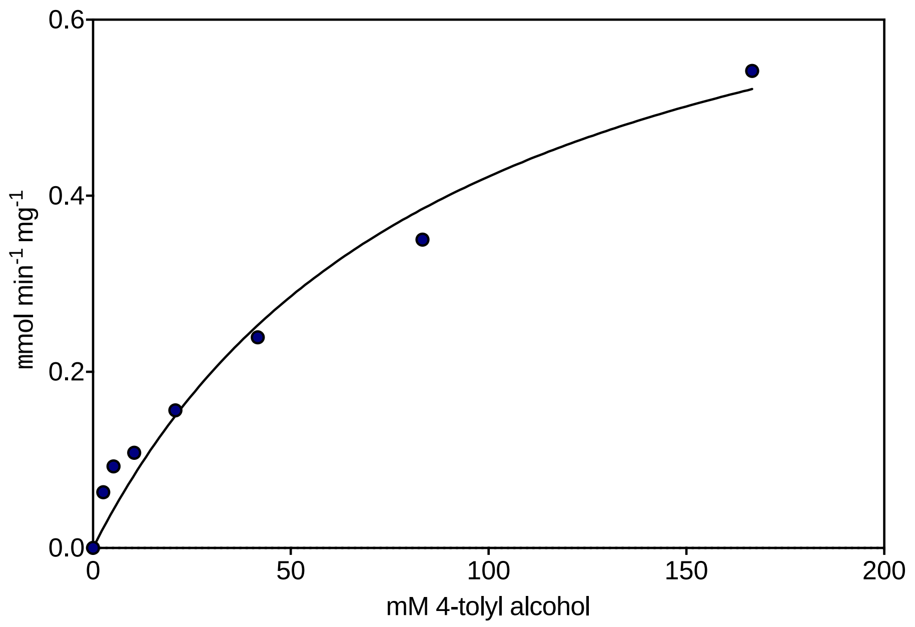

Supplementary Figure 29 - The kinetics of apADH in the oxidative direction at 70 °C, pH 7.5 with varying concentrations of 4-tolyl alcohol.
Data have been fitted to the Michaelis-Menten equation.

**Supplementary Figure 30 - Parameters calculated fitting kinetics of apADH in the oxidative direction at 70 °C, pH 7.5, with varying concentrations of NAD^+, to the Michaelis-Menten equation.** As substrate concentration could not be taken high enough to calculate an accurate K_M or V_{MAX} this data was not used. An approximate K_M of 100 mM was used and kcat in the oxidative direction assumed to be approximately equal to the forward.	
**Best-fit values**	
V_{MAX} (µmol / min / mg)	0.80
K_M *p*-tolyl alcohol (mM)	90
**Std. Error**	
V_{MAX} (µmol / min / mg)	0.06
K_M *p*-tolyl alcohol (mM)	15

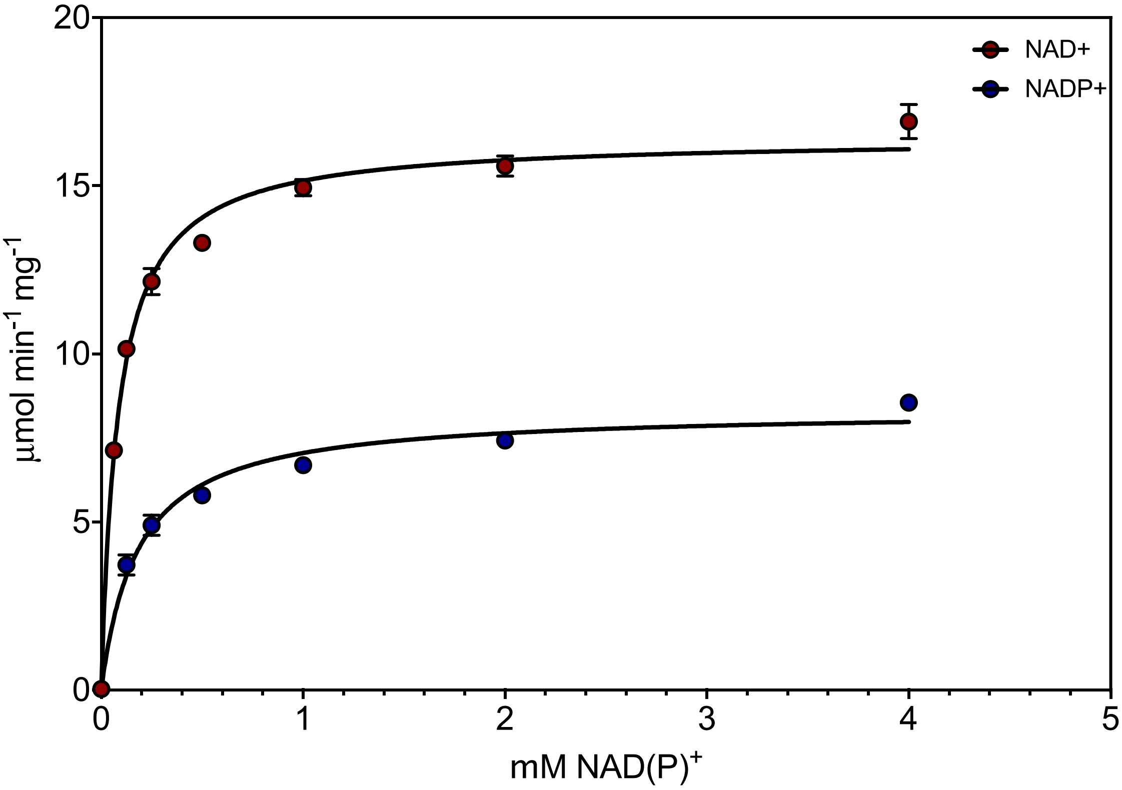

Supplementary Figure 31 – The kinetics of PTDH with varying concentrations of NADP⁺ and NAD⁺. Data have been fitted to the Michaelis-Menten equation.

**Supplementary Figure 32 - Parameters calculated fitting kinetics of PTDH with varying concentrations of NADP⁺ and NAD⁺ to the Michaelis-Menten equation.**		
**Best-fit values**	**NADP⁺**	**NAD⁺**
V_{MAX} (µmol / min / mg)	8.3	16.4
K_M NAD(P)⁺(µM)	180	85
**Std. Error**		
V_{MAX} (µmol / min / mg)	0.2	0.2
K_M 4-tolyl alcohol (mM)	20	5

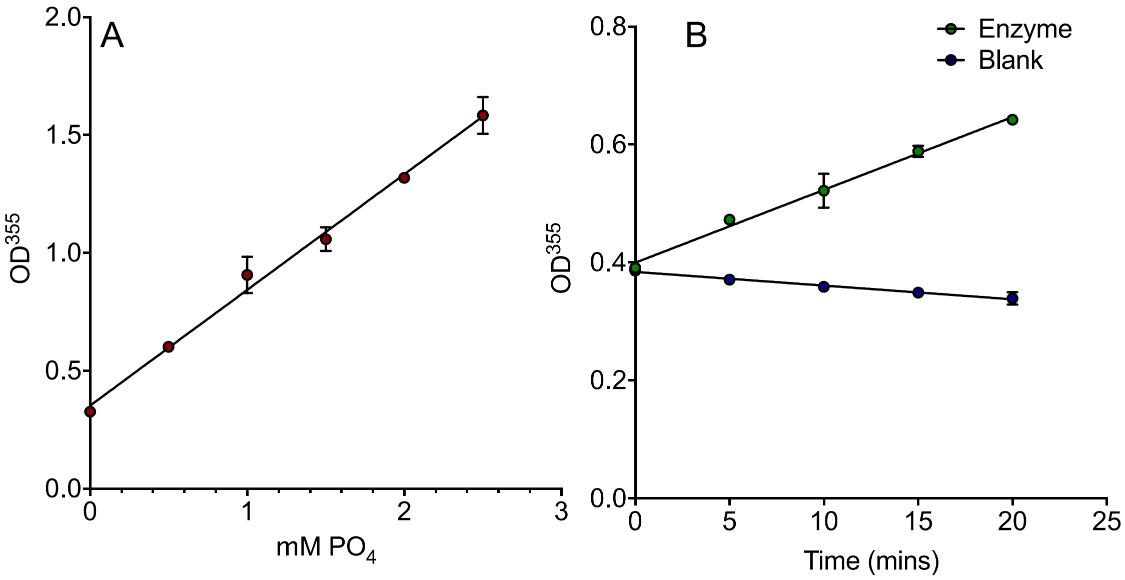

Supplementary Figure 33 – Determining k_{cat} for ttPPiase

A: A standard curve of phosphate concentration vs OD 335 nm was determined for the assay.

B: The rate of phosphate production was determined (green circles), along with a blank rate (blue circles). Rate of blank subtracted phosphate production was calculated from the standard curve, from which k_{cat} was calculated.

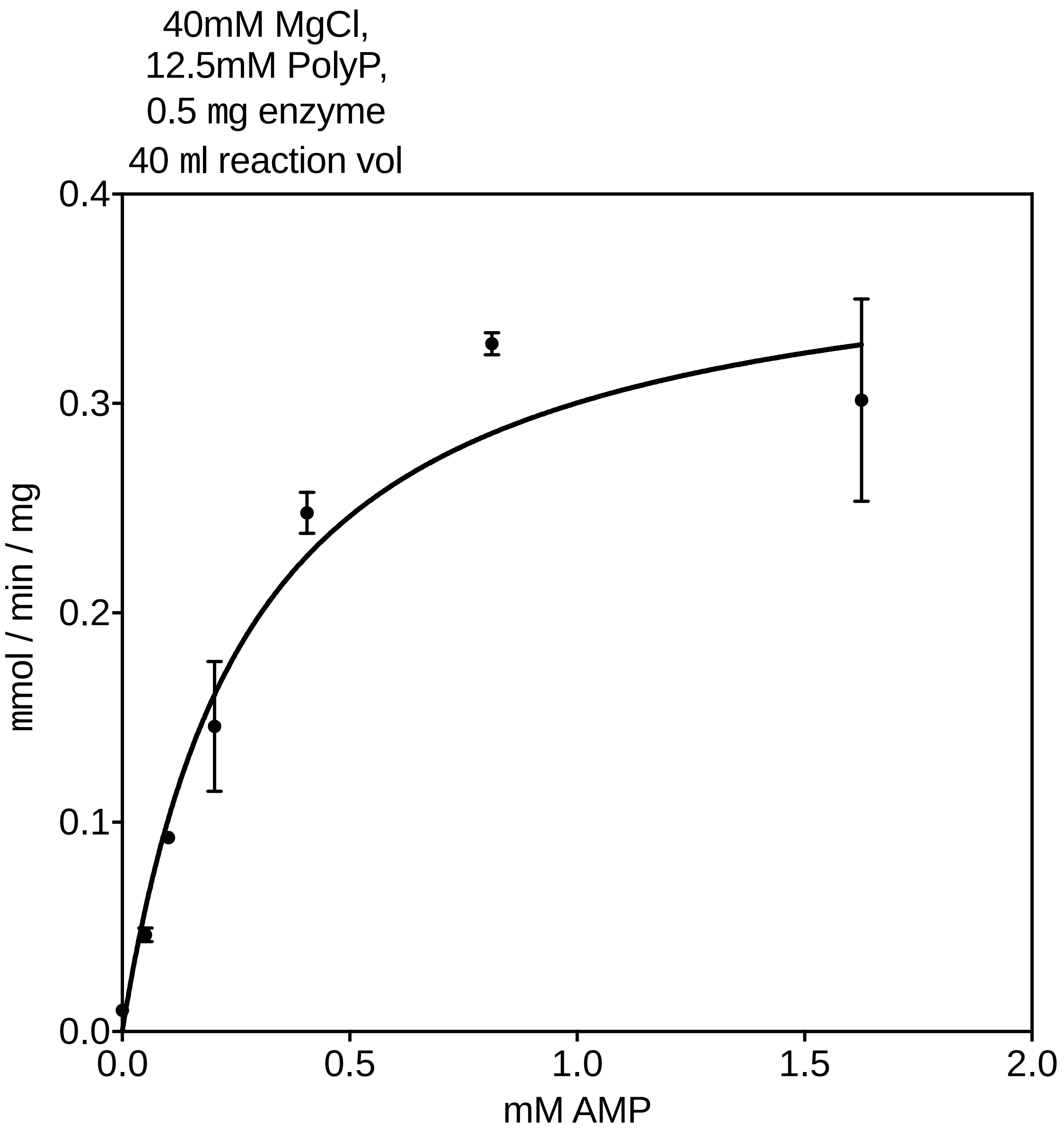

Supplementary Figure 34 – The kinetics of tnPAP with varying concentrations of AMP
Data fitted to the Michaelis-Menten equation.

**Supplementary Figure 35 - Parameters calculated fitting kinetics of tnPAP with varying concentrations of AMP to the Michaelis-Menten equation.**	
**Best-fit values**	
V_{MAX} (µmol / min / mg)	0.39
K_M AMP (mM)	0.28
**Std. Error**	
V_{MAX} (µmol / min / mg)	0.03
K_M AMP (mM)	0.05

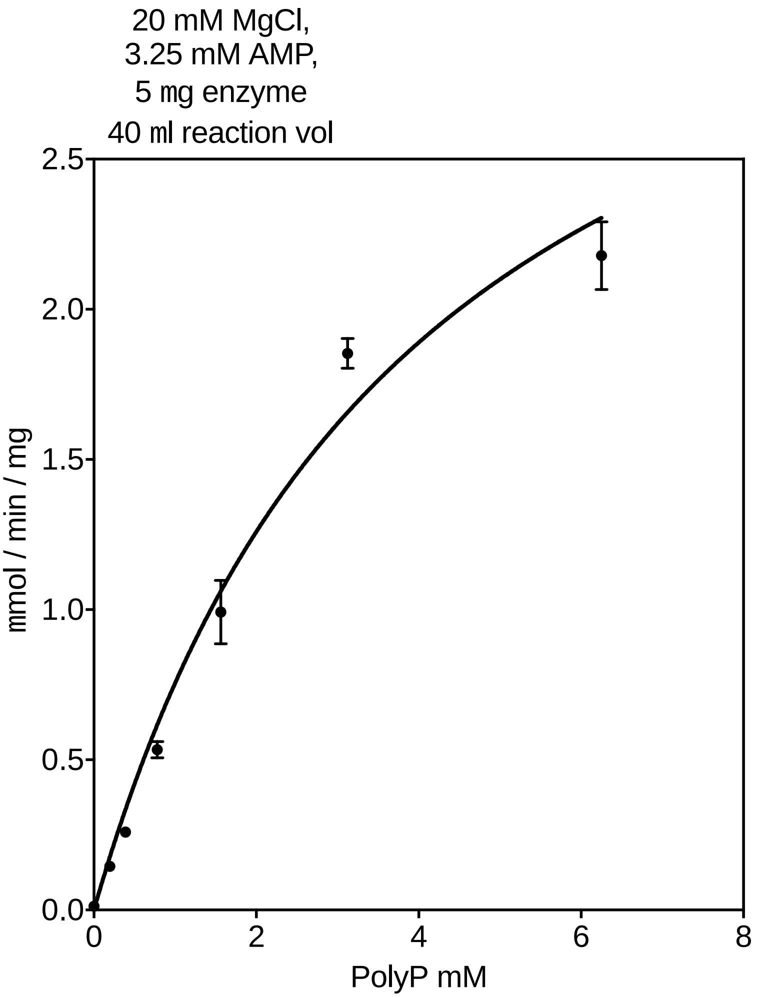

Supplementary Figure 36 - The kinetics of tnPAP with varying concentrations of Polyphosphate (PolyP)
Data have been fitted to the Michaelis-Menten equation.

Supplementary Figure 37 - Parameters calculated fitting kinetics of tnPAP with varying concentrations of polyphosphate to the Michaelis-Menten equation.

Best-fit values	
Vmax	3.781
Km	4.004
Std. Error	
Vmax	0.3454
Km	0.6808

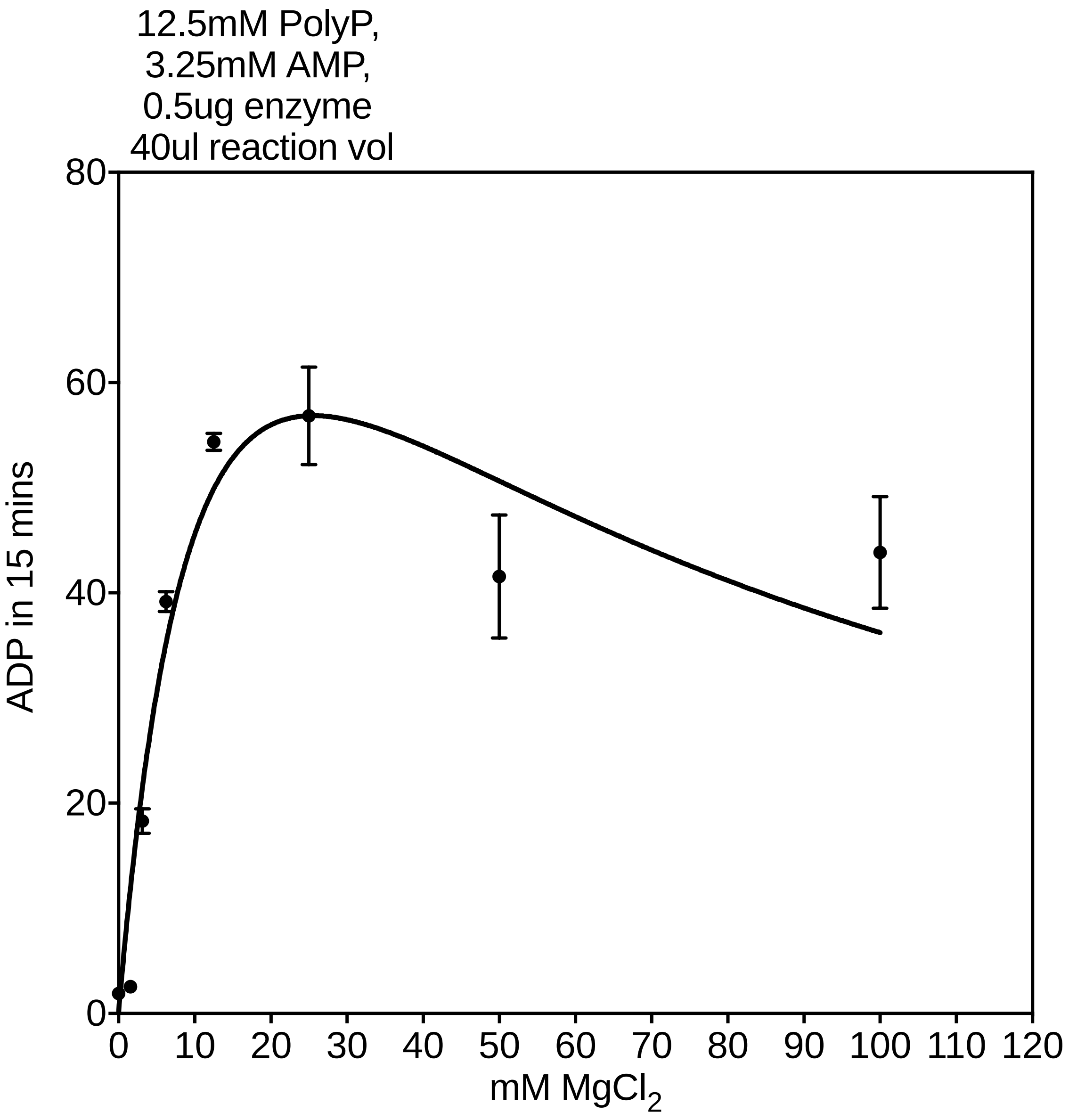

Supplementary Figure 38 - The kinetics of tnPAP with varying concentrations of $MgCl_2$
Data have been fitted to the Michaelis-Menten equation with substrate inhibition.

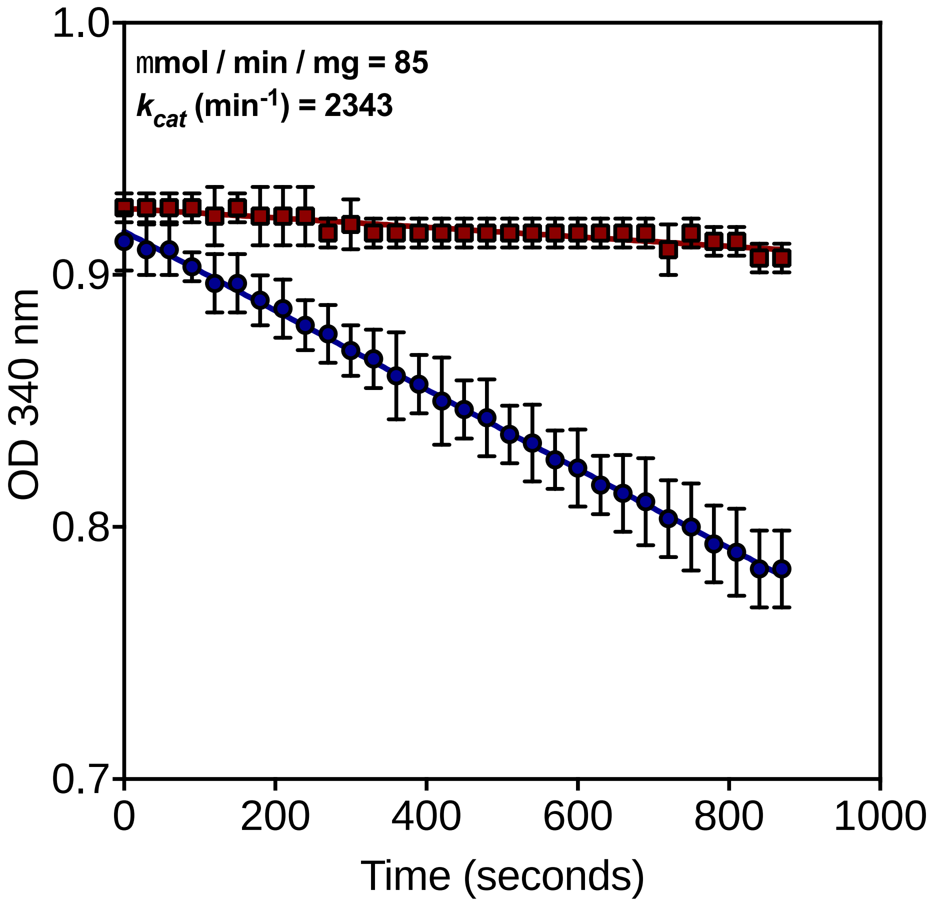

Supplementary Figure 39 – tnAK – CAR coupled assay to estimate *k*$_{cat}$
tnAK was coupled to a CAR enzyme in order to estimate its k_{cat} in the ADP to ATP direction. The rate obtained was significantly slower than previously reported. The k_{cat} for the reverse reaction was adjusted relative. Red circles show the blank rate, blue squares show the rate with tnAK.

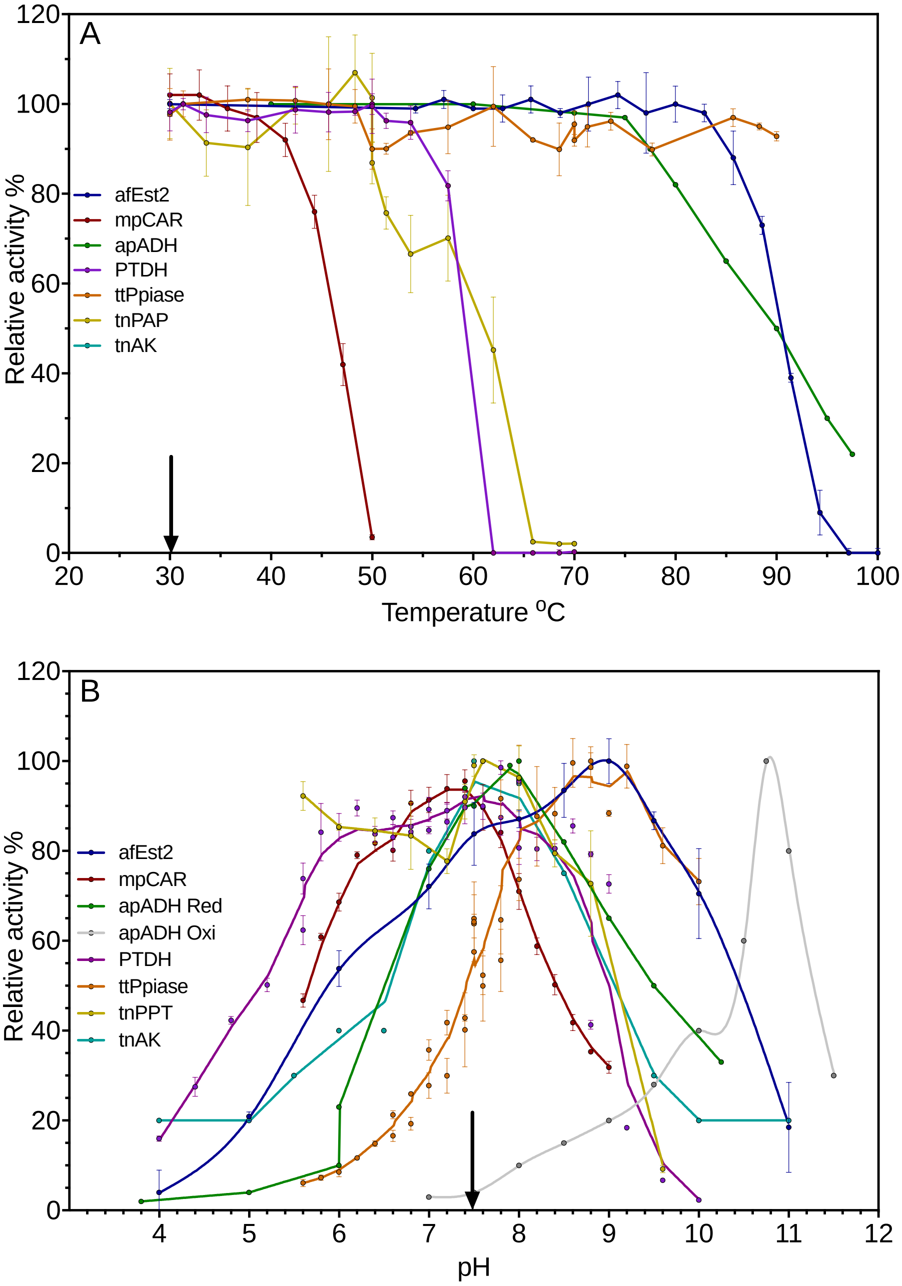

Supplementary Figure 40 - The operational window for temperature (A) and pH (B) for the seven enzyme reaction

A. Residual relative activity after incubation at various temperatures for 30 minutes. The selected operational temperature is shown by the black arrow.
B. Relative activity at various pH values. Values are relative to the maximum activity in each case. The selected operational pH is shown by the black arrow.

Data show the mean of three experimental replicates for each point, with error bars representing ±SD. Data for afEst2(42), mpCAR(15), apADH(46) and tnAK (45) were adapted from previous work.

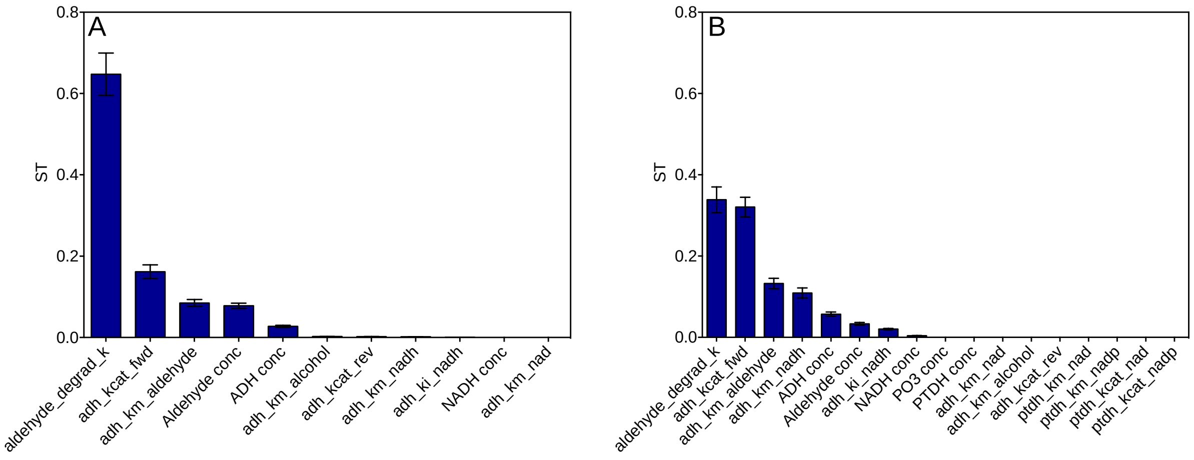

Supplementary Figure 41 – Sensitivity analysis of apADH and apADH-PTDH modelled reactions.
The total sensitivity indices (ST) are shown which take into account 1st order and all other interactions. Sensitivity is in reference to the uncertainty in the final *p*-tolyl alcohol concentration. Error bars show the 95 % confidence intervals. The sum of all sensitivity indices' should equal 1.
A: Sensitivity analysis of apADH only reaction, figure 4A.
B: Sensitivity analysis of the apADH-PTDH reaction, figure 4B.

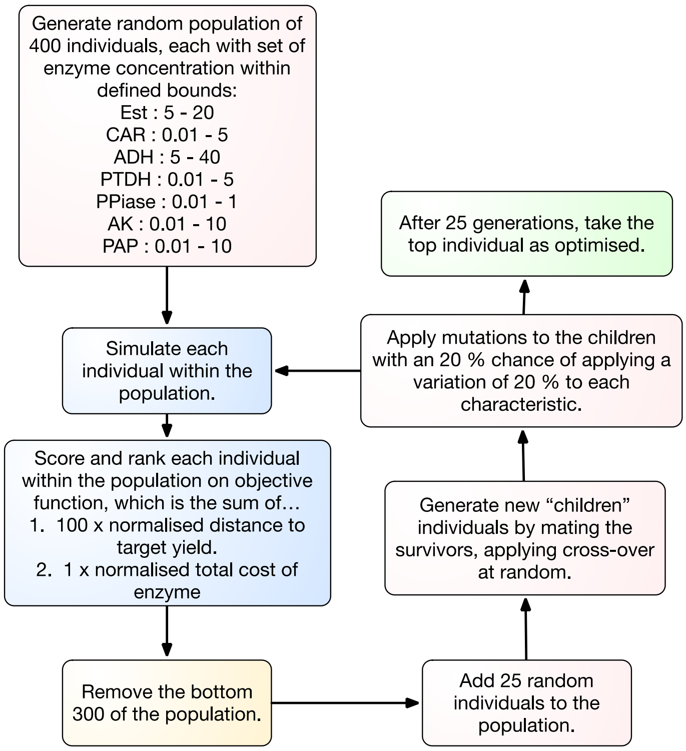

Supplementary Figure 42 - Flow diagram for genetic algorithm used to optimize the reaction
Our custom built genetic algorithm was used in the optimization of a batch reaction, minimizing total enzyme cost whilst achieving a target yield of 90 % or above. The flow diagram describes the steps the genetic algorithm carries out to reach this goal.

Supplementary Modelling: Alternative model incorporating magnesium.

Following the completion of the rest of this study, we investigated a more complex model incorporating the binding of magnesium to various species. The rate equations for the adenylate kinase (AK) could then be amended to match the random sequential mechanism described in previous studies [2]. Dissociation constants (K_D) were found for magnesium binding to ATP, ADP and PP_I, which were used to estimate k_1 (or k_{on}) and k_{-1} (or k_{off}) for the binding and unbinding of magnesium from these species. To do this we estimated k_1 as between 0.001 – 10 $\mu M^{-1}\ s^{-1}$, and used K_D to calculate k_{-1} (Supplementary Table 3).

Comparisons of this new model with the original version show only minor changes in the predictions (Supplementary Figures 43 to 46). Magnesium chelation by polyphosphate has been shown to be an issue by others [3], and this new model likely still does not account for this properly. Furthermore, our optimization was carried out using the original model. For these reasons we opted to use our original model in the main study.

Changes to rate equations in the original model

Rate equations featuring ATP now utilize MgATP. PAP takes MgPolyP. PPiase takes $MgPP_i$. Adenylate kinase is now modelled as a pair of bi random sequential equations.

$$r3 = c_{CAR} \cdot k_{cat}^{CAR} \cdot \frac{c_{Acid} \cdot c_{MgATP} \cdot c_{NADPH}}{(K_I^{MgATP} \cdot K_M^{Acid}) + (K_M^{NADPH} \cdot c_{MgATP} \cdot c_{Acid}) + (K_M^{Acid} \cdot c_{MgATP} \cdot c_{NADPH}) + (K_M^{MgATP} \cdot c_{Acid} \cdot c_{NADPH}) + (c_{MgATP} \cdot c_{Acid} \cdot c_{NADPH})}$$

$$r8 = c_{PPiase} \cdot k_{cat}^{PPiase} \cdot \frac{c_{MgPPi}}{c_{MgPPi} + K_M^{MgPpi}}$$

$$r9 = c_{PPT} \cdot k_{cat}^{PPTFwd} \cdot \frac{c_{MgPolyP}}{c_{MgPolyP} + K_M^{MgPolyP}} \cdot \frac{c_{AMP}}{c_{AMP} + K_M^{AMP}}$$

$$r11 = c_{AK} \cdot k_{cat}^{AK-Fwd} \cdot \frac{c_{ADP} \cdot c_{MgADP}}{(K_I^{ADP} \cdot K_M^{MgADP}) + (K_M^{MgADP} \cdot c_{ADP}) + (K_M^{ADP} \cdot c_{MgADP}) + (c_{ADP} \cdot c_{MgADP})}$$

$$r12 = c_{AK} \cdot k_{cat}^{AK-Rev} \cdot \frac{c_{AMP} \cdot c_{MgATP}}{(K_I^{AMP} \cdot K_M^{MgATP}) + (K_M^{MgATP} \cdot c_{AMP}) + (K_M^{AMP} \cdot c_{MgATP}) + (c_{AMP} \cdot c_{MgATP})}$$

New rate equations in this alternative model

$$r15 = (c_{ATP} \cdot c_{Mg} \cdot k_1^{ATPMg}) - (c_{MgATP} \cdot k_{-1}^{ATPMg})$$
$$r16 = (c_{ADP} \cdot c_{Mg} \cdot k_1^{ADPMg}) - (c_{MgADP} \cdot k_{-1}^{ADPMg})$$
$$r17 = (c_{PPi} \cdot c_{Mg} \cdot k_1^{PPiMg}) - (c_{MgPPi} \cdot k_{-1}^{PPiMg})$$
$$r18 = (c_{PolyP} \cdot c_{Mg} \cdot k_1^{PolyPMg}) - (c_{MgPolyP} \cdot k_{-1}^{PolyPMg})$$

Modelling the binding of magnesium to polyphosphate in this way assumes one magnesium ion binds one PO_3 unit on the polyphosphate, which is likely incorrect.

New parameters

Parameters present in the original rate equations and corresponding to magnesium bound species in this model, remain the same. Where possible, new parameters were taken from the literature ± 50%, as referenced. Where no reference is given the parameter bounds are estimates.

Supplementary Table 3– Parameters in the alternative model	
**Parameter**	**Upper and lower bounds**
**Adenylate Kinase**	
K_I^{ADP}	1650 – 4950 μM [2]
K_I^{AMP}	455 – 1365 μM [2]
**ATP - Magnesium binding equilibrium**	
k_{ATPMg}^D	17.5 - 52.5 μM [4]

k^{1}_{ATPMg}	0.001 – 10 μM^{-1} s^{-1}
k^{-1}_{ATPMg}	$k^{1}_{ATPMg} \cdot k^{D}_{ATPMg}$
**ADP - Magnesium binding equilibrium**	
k^{D}_{ADPMg}	335 – 1005 μM [4]
k^{1}_{ADPMg}	0.001 – 10 μM^{-1} s^{-1}
k^{-1}_{ADPMg}	$k^{1}_{ADPMg} \cdot k^{D}_{ADPMg}$
**PPi - Magnesium binding equilibrium**	
k^{D}_{PPiMg}	25 - 75 μM [5]
k^{1}_{PPiMg}	0.001 – 10 μM^{-1} s^{-1}
k^{-1}_{PPiMg}	$k^{1}_{PPiMg} \cdot k^{D}_{PPiMg}$
**PolyP - Magnesium binding equilibrium**	
$k^{D}_{PolyPMg}$	10 - 500 μM
$k^{1}_{PolyPMg}$	0.001 – 10 μM^{-1} s^{-1}
$k^{-1}_{PolyPMg}$	$k^{1}_{PolyPMg} \cdot k^{D}_{PolyPMg}$

Additional differential equations

Additional differential equations were used to model the equilibrium between the free and magnesium bound species for ATP, ADP, PPi and PolyP, using the new rate equations r15 to r18.

For example:

$$\frac{dATP}{dt} = -r15$$

$$\frac{dMgATP}{dt} = r15 - r4 + r11 - r12$$

See supplementary table 2 for a list of differential equations used in the original model.

mpCAR-ttPPiase-tnPAP-tnAK-PTDH

Comparison of the original model output to the alternative model incorporating magnesium.

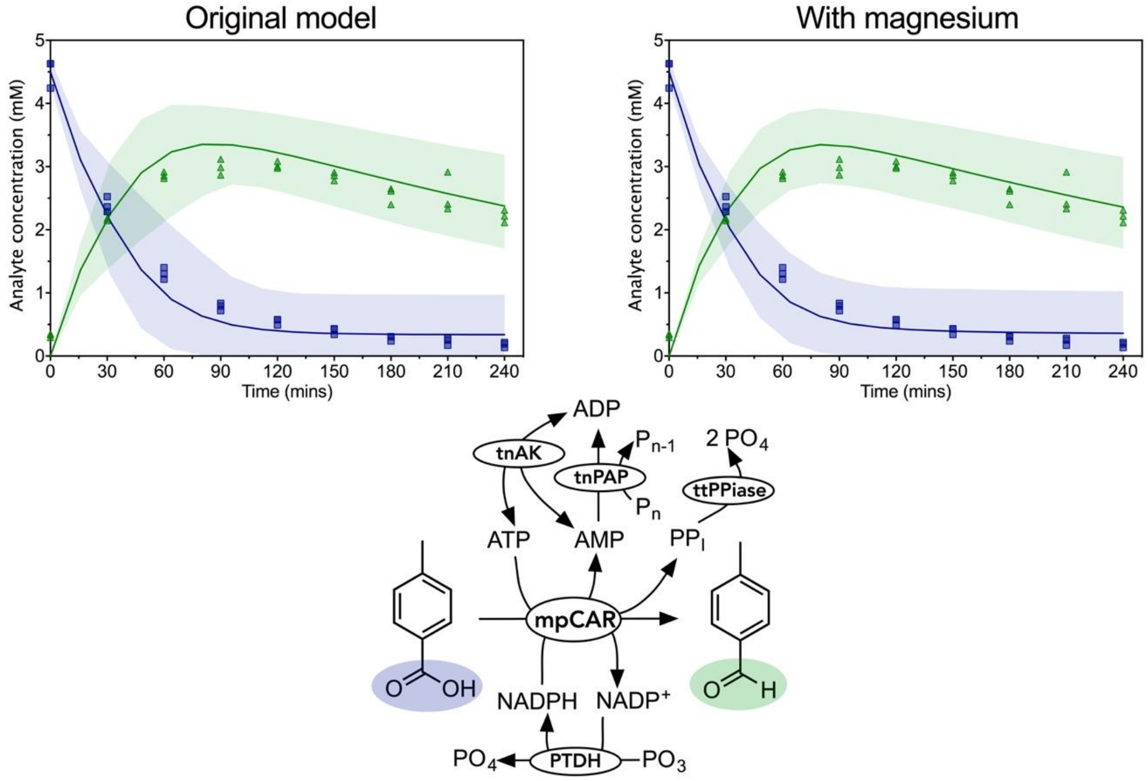

Supplementary Figure 43 – Comparison of original model and the alternative model for the mpCAR-ttPPiase-tnPAP-tnAK-PTDH reaction, featuring magnesium binding equilibria for ATP, ADP, PPi and PolyP and other amended rated equations.

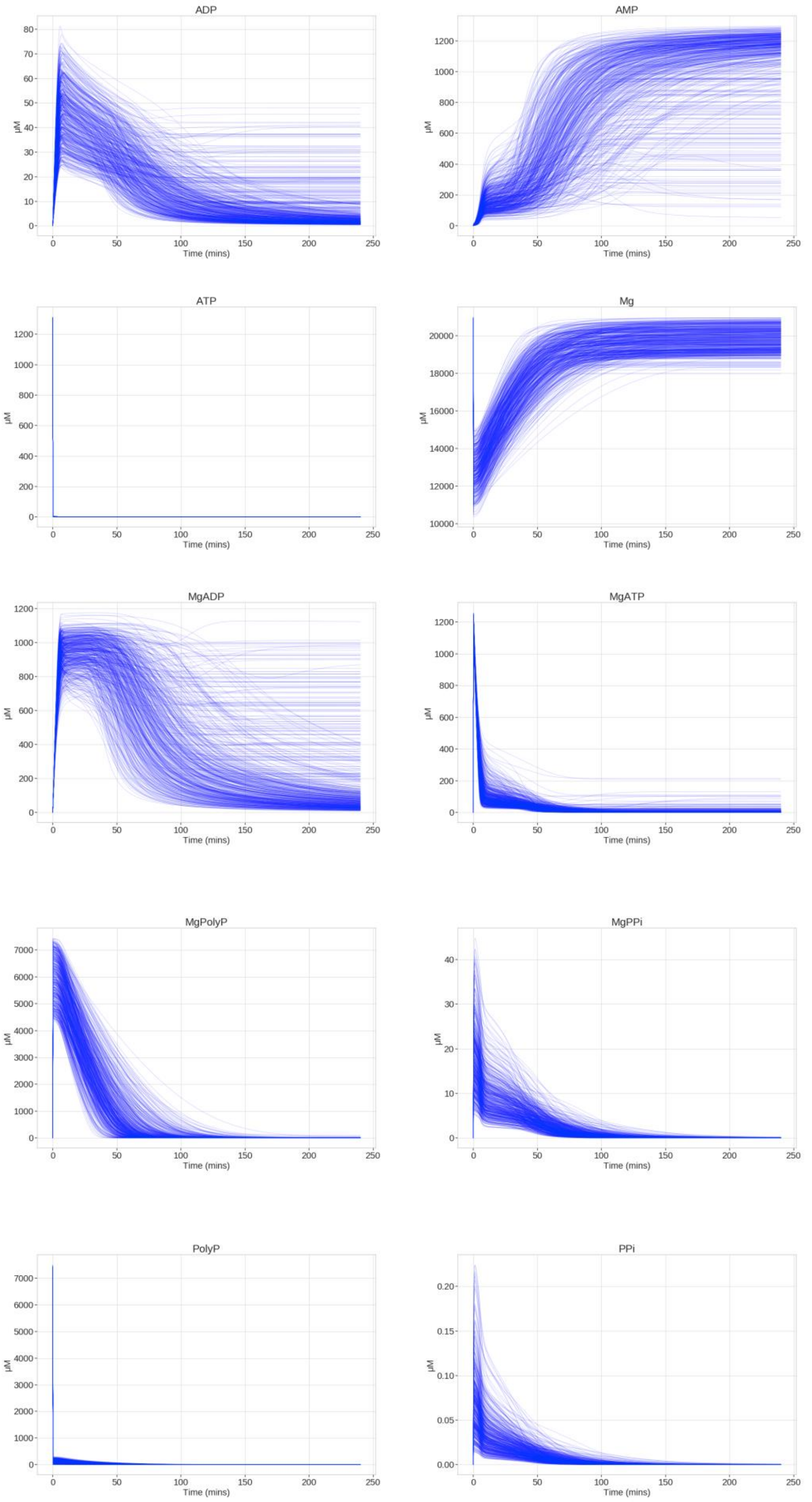
ADP
AMP
ATP
Mg
MgADP
MgATP
MgPolyP
MgPPi
PolyP
PPi
µM
Time (mins)

Supplementary Model Figure 44 – mpCAR-ttPPiase-tnPAP-tnAK-PTDH model predictions for free and magnesium bound species during the course of the reaction. Each line represents a single model run with sampled parameters, with a total of 500 models run.

Unoptimized complete model comparison

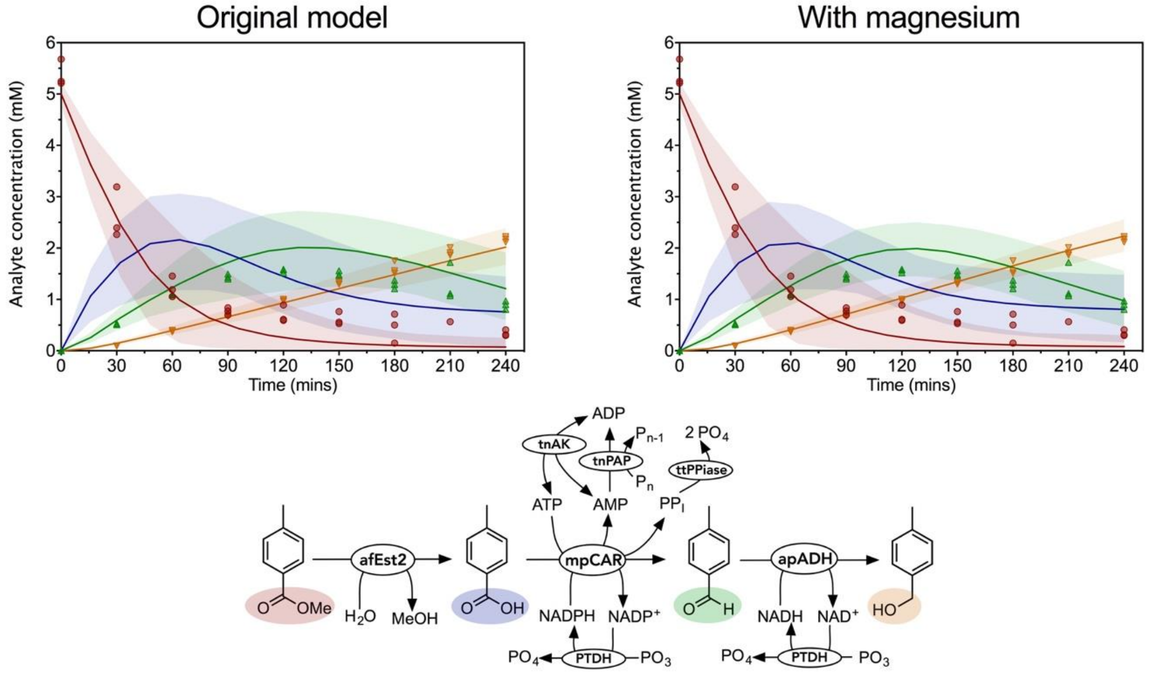

Supplementary Model Figure 45 – Comparison of original model and the alternative model for the complete, unoptimized reaction, featuring magnesium binding equilibrium for ATP, ADP, PPi and PolyP and other amended rated equations.

Optimized complete model comparison

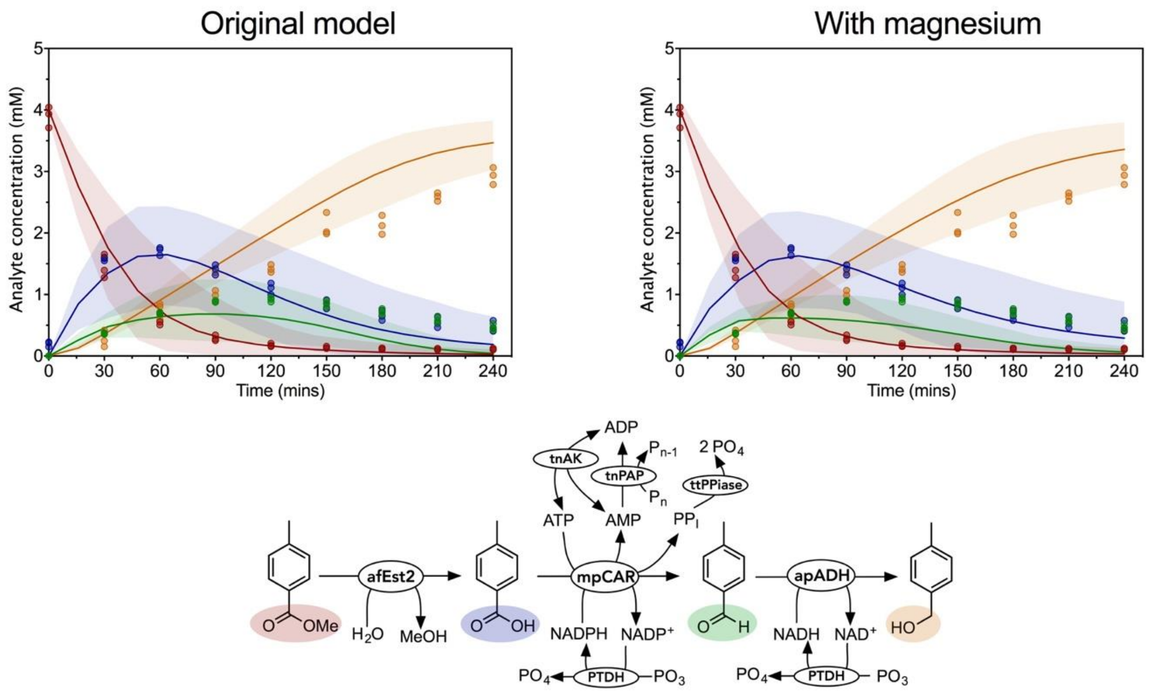

Supplementary Model Figure 46 – Comparison of original model and the alternative model for the complete, optimized reaction, featuring magnesium binding equilibrium for ATP, ADP, PPi and PolyP and other amended rated equations.

References

[1] A. Kümmel, S. Panke, M. Heinemann, *BMC Bioinformatics* **2006**, *7*, 1–12.

[2] X. R. Sheng, X. Li, X. M. Pan, *J. Biol. Chem.* **1999**, *274*, 22238–22242.

[3] G. A. Strohmeier, I. C. Eiteljörg, A. Schwarz, M. Winkler, *Chem. - A Eur. J.* **2019**, 6119–6123.

[4] E. Gout, F. Rebeille, R. Douce, R. Bligny, *Proc. Natl. Acad. Sci.* **2014**, *111*, E4560–E4567.

[5] S. Uribe, P. Rangel, J. P. Pardo, L. Pereira Da Silva, *Eur. J. Biochem.* **1993**, *217*, 657–660.
