# Supplementary material for: Engineering a Seven Enzyme Biotransformation using Mathematical Modelling and Characterized Enzyme Parts
Source: ChemCatChem. 2019 Jul 4;11(15):3474–89. doi: 10.1002/cctc.201900646 (PMC6774274; doi:10.1002/cctc.201900646)
Supplement: Supplementary file 2 — Supplementary [file CCTC-11-3474-s002.zip › Supplementary Data for Uploading/Modelling/Modelling 1.6/License.txt]

Copyright 2017 William FinniganPermission is hereby granted, free of charge, to any person obtaining a copy of this software and associated documentation files (the "Software"), to deal in the Software without restriction, including without limitation the rights to use, copy, modify, merge, publish, distribute, sublicense, and/or sell copies of the Software, and to permit persons to whom the Software is furnished to do so, subject to the following conditions:The above copyright notice and this permission notice shall be included in all copies or substantial portions of the Software.THE SOFTWARE IS PROVIDED "AS IS", WITHOUT WARRANTY OF ANY KIND, EXPRESS OR IMPLIED, INCLUDING BUT NOT LIMITED TO THE WARRANTIES OF MERCHANTABILITY, FITNESS FOR A PARTICULAR PURPOSE AND NONINFRINGEMENT. IN NO EVENT SHALL THE AUTHORS OR COPYRIGHT HOLDERS BE LIABLE FOR ANY CLAIM, DAMAGES OR OTHER LIABILITY, WHETHER IN AN ACTION OF CONTRACT, TORT OR OTHERWISE, ARISING FROM, OUT OF OR IN CONNECTION WITH THE SOFTWARE OR THE USE OR OTHER DEALINGS IN THE SOFTWARE.
